# Curcumin alleviates metabolic dysfunction-associated steatotic liver disease via gut microbiota-dependent epigenetic alterations

**DOI:** 10.1186/s43556-026-00606-1

**Published:** 2026-10-16

**Authors:** Yong Zhu, Jinhu Ma, Danhong Wang, Mingzhu Cui, Jin Peng, Xuejin Lu, Xingyue Pei, Yi Wang, Jing Lv, Yuxuan Yan, Wenhao Ma, Mingtao Zhao, Shulei You, Jie Ma, Yunshu Tang, Yaling Zhu

**Affiliations:** 1 Department of Pathophysiology, College of Basic Medical Science, Anhui Medical University, Hefei, Anhui 230032 China; 2 Department of Hepatobiliary Surgery, State Key Laboratory of Immune Response and Immunotherapy, Centre for Leading Medicine and Advanced Technologies of IHM, The First Affiliated Hospital of USTC, Division of Life Sciences and Medicine, University of Science and Technology of China, Hefei, Anhui 230026 China; 3 Laboratory Animal Research Center, College of Basic Medical Science, Anhui Medical University, Hefei, Anhui 230032 China

**Keywords:** Curcumin, MASLD, Multi-omics, Gut microbiota, H3K27ac, Epigenetics

## Abstract

Metabolic dysfunction-associated steatotic liver disease (MASLD) is increasingly prevalent, yet effective therapies remain limited. Curcumin (Cur), a polyphenol with well-documented metabolic benefits, shows therapeutic promise in MASLD, but its efficacy and underlying mechanisms remain incompletely understood. A meta-analysis of seven clinical studies indicated that Cur supplementation reduced serum aminotransferase levels, supporting its potential clinical benefit. In high-fat diet (HFD)-fed rats, Cur reduced hepatic lipid accumulation and metabolic dysfunction, whereas these effects were markedly weakened in antibiotic-induced pseudo-germ-free rats, indicating microbiota dependence. Fecal microbiota transplantation from Cur-treated donors transferred these metabolic and histological benefits to antibiotic-conditioned recipients. Fecal 16S rRNA sequencing identified *Clostridium_sensu_stricto_1* (*C. sensu*) as a Cur-suppressed, pro-steatotic taxon, and targeted reconstitution of this taxon abolished Cur-mediated protection. Untargeted hepatic metabolomics and liquid chromatography–mass spectrometry identified kynurenine (KYN) as a *C. sensu*-derived metabolite and showed that Cur reduced hepatic KYN accumulation. Integrated H3K27ac ChIP-seq, bulk RNA-seq, and single-cell analyses pinpointed hepatocyte-enriched *CEBPB* as a key epigenetically regulated effector. Luciferase reporter assays, ChIP-qPCR, and gain- and loss-of-function experiments showed that KYN activated the aryl hydrocarbon receptor (AHR), increased H3K27ac enrichment at the *Cebpb* locus, and induced *Cebpb* transcription. *CEBPB* knockdown blocked KYN-induced PI3K-AKT activation and lipid accumulation, whereas in vivo KYN supplementation largely reversed Cur-mediated protection. Collectively, this study reveals a novel gut-liver axis in MASLD pathogenesis, demonstrating that Cur alleviates hepatic steatosis by targeting the microbiota-KYN-AHR-H3K27ac-*Cebpb* epigenetic axis, positioning Cur supplementation and KYN modulation as promising therapeutic strategies for MASLD intervention.

**Supplementary Information:**

The online version contains supplementary material available at https://doi.org/10.1186/s43556-026-00606-1.

## Introduction

Metabolic dysfunction-associated steatotic liver disease (MASLD), characterized by excessive hepatic lipid accumulation in the setting of metabolic disorders such as obesity, insulin resistance, and dyslipidemia, has become the most prevalent chronic liver disease worldwide [1]. The global surge in metabolic syndrome has positioned MASLD as a major precursor to end-stage liver diseases, including cirrhosis and hepatocellular carcinoma [2, 3]. Despite its growing disease burden, effective pharmacological therapies for MASLD remain limited because of its multifactorial pathogenesis involving multiple organs and signaling pathways. Curcumin (Cur), a plant-derived polyphenol from traditional Chinese medicine, has gained extensive attention for its diverse pharmacological activities, including anti-inflammatory, antioxidant, anticancer, and hepatoprotective effects [4, 5]. However, the mechanistic basis for its hepatoprotective effects, particularly regarding gut microbiota modulation and epigenetic regulation, requires systematic investigation.

The gut-liver axis, mediated by the enterohepatic circulation through the portal vein system, significantly influences the pharmacological response to medications and the progression of metabolic disorders [6]. Accumulating evidence identifies gut microbiota dysbiosis as a key driver of MASLD initiation and progression. Mechanistically, microbial dysbiosis can directly compromise intestinal barrier integrity, facilitating bacterial translocation and subsequent inflammation, or indirectly modulate host metabolism through microbiota-derived metabolites [7–10]. Given that Cur is administered orally, it is likely to exert part of its hepatoprotective effects through modulation of the gut microbiota. This suggests that targeting the gut microbiota with Cur could offer novel therapeutic strategies for MASLD management.

Emerging evidence indicates that gut dysbiosis and microbial metabolites (e.g., short-chain fatty acids (SCFAs) and bile acids) can induce hepatic epigenetic modifications (DNA methylation, histone acetylation), thereby contributing to liver disease progression. Ha et al*.* demonstrated that alterations in gut microbiota composition and microbial metabolites induce hepatic epigenetic remodeling, while dysregulated gene expression, in turn, reshapes the composition and function of the gut microbiota and its derived metabolites [11]. Our previous work demonstrated that MASLD was accompanied by widespread increases in H3K27ac occupancy and extensive transcriptomic remodeling, which increased the expression of *TDO2* (the rate-limiting enzyme catalyzing the conversion of tryptophan to kynurenine (KYN)) and enhanced kynurenine biosynthesis, and consequently accelerated macrophage M1 polarization and disease progression [12]. Furthermore, Liu et al*.* provided evidence that Cur can exert epigenetic regulatory effects by modulating H3K27ac [13]. These findings suggest that Cur may exert its effects by remodeling the gut microbiota-metabolite-epigenetics axis, though the precise mechanisms require further elucidation.

Herein, we integrated multi-omics approaches, including 16S rRNA microbiomics, metabolomics, H3K27ac epigenomics, bulk transcriptomics, and single-cell transcriptomics, to elucidate the mechanisms underlying the therapeutic effects of Cur on MASLD, with a particular focus on gut microbiota and H3K27ac-mediated epigenetic regulation. Notably, we demonstrated that Cur ameliorates hepatic steatosis in a gut microbiota-dependent manner, specifically by reducing the abundance of the pathogenic *taxon Clostridium sensu stricto 1 (C. *sensu). Mechanistically, the gut microbiota-derived metabolite KYN bound to the aryl hydrocarbon receptor (AHR), thereby enhancing H3K27ac enrichment at the *Cebpb* locus, upregulating *Cebpb* expression, activating PI3K-AKT signaling, and ultimately promoting hepatic steatosis. Taken together, these findings show that Cur ameliorates MASLD progression by targeting the gut microbiota-KYN axis to epigenetically suppress *Cebpb* transcription, thereby providing a novel mechanistic basis for MASLD treatment.

## Results

### Cur alleviated HFD-induced hepatic steatosis and injury in a gut microbiota-dependent manner

**Fig. 1:**
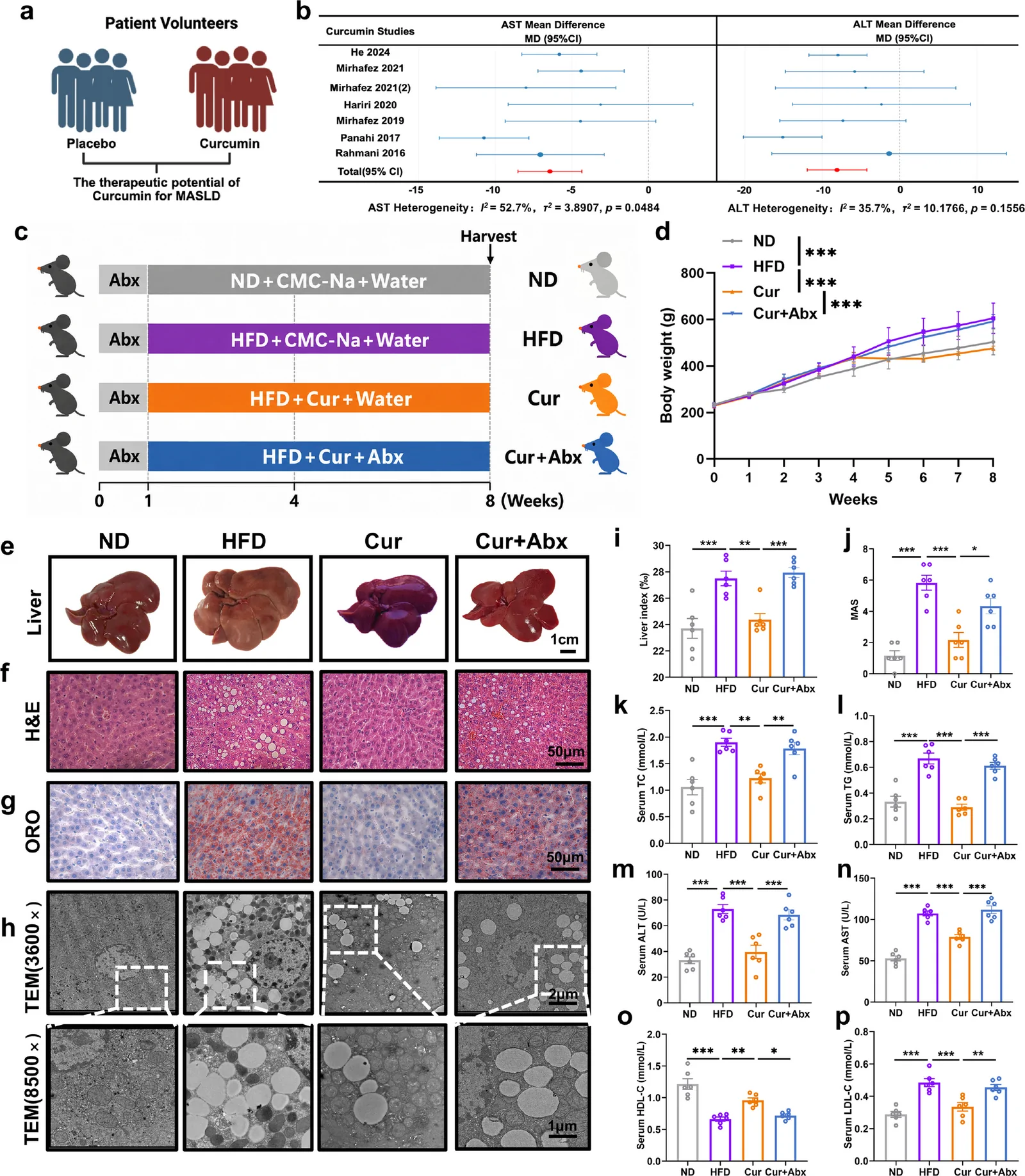
Gut microbiota plays a critical role in the therapeutic effects of Cur on HFD-induced hepatic steatosis. **a** Schematic overview of the included patient cohorts receiving placebo or Cur treatment (7 studies, *n* = 467 participants). **b** Forest plots showing pooled mean differences (MDs) with 95% confidence intervals (CIs) for AST and ALT levels following curcumin supplementation (7 studies, *n* = 467 participants). “Mirhafez 2021 (1)” and “Mirhafez 2021 (2)” represent two independent cohorts reported in the same publication and were analyzed separately. Pooled effect sizes were calculated using a random-effects model. Between-study heterogeneity was assessed using Cochran’s Q test and quantified by the I^2^ statistic, while between-study variance (τ^2^) was estimated using the restricted maximum likelihood (REML) method. For AST, heterogeneity was moderate (I^2^ = 52.7%, τ^2^ = 3.8907, *P* = 0.0484). For ALT, heterogeneity was low-to-moderate (I^2^ = 35.7%, τ^2^ = 10.1766, *P* = 0.1556). Red circles indicate pooled effect estimates and horizontal lines represent 95% CIs. **c** Schematic representation of the experimental design (*n* = 6). **d** Body weight of rats fed a normal diet (ND group), high-fat diet (HFD group), HFD with Cur (Cur group), and HFD with Cur and antibiotics (Cur + Abx group), *n* = 6, two-way ANOVA followed by Bonferroni post-test. **e** Representative images of liver morphology from rats in ND, HFD, Cur, and Cur + Abx groups (scale bar, 1 cm, *n* = 6). **f** Histopathological sections of liver tissue stained with hematoxylin and eosin (scale bar, 50 μm, *n* = 6). **g** Oil Red O (ORO) staining of the livers from the indicated groups (scale bar, 50 μm, *n* = 6). **h** Transmission electron microscopy (TEM) images of hepatocytes from ND, HFD, Cur, and Cur + Abx groups at 3600 × magnification (upper, scale bar, 2 μm) and 8500 × magnification (lower, scale bar, 1 μm) (*n* = 6). **i** The liver index (ratio of liver weight to body weight) for ND, HFD, Cur, and Cur + Abx rats (*n* = 6). **j** MASLD activity score (MAS) in the indicated groups (*n* = 6). **k-p** Levels of serum TC (**k**), serum TG (**l**), serum ALT (**m**), serum AST (**n**), serum HDL-C (**o**), and serum LDL-C (**p**) in the indicated groups (*n* = 6). All data are represented as the mean ± SD. *P* values were calculated by two-way ANOVA followed by Bonferroni post-test in (**d**), others with one-way ANOVA followed by Tukey’s test. *P < 0.05, **P < 0.01, ***P < 0.001. ALT: alanine aminotransferase, AST: aspartate aminotransferase, TG: triglycerides, TC: total cholesterol, HDL-C: high-density lipoprotein cholesterol, LDL-C: low-density lipoprotein cholesterol

To investigate the therapeutic potential of Cur in MASLD, we first performed a meta-analysis using data extracted from seven previously published clinical studies involving MASLD/NAFLD patients treated with Cur [14–20], assessing the effects of Cur versus control on two key liver enzymes, aspartate aminotransferase (AST) and alanine aminotransferase (ALT). The forest plots collectively indicated that Cur may have a therapeutic effect on liver function in MASLD patients, as evidenced by significant mean differences (MDs) with their 95% confidence intervals (CIs) for individual studies and the overall effect on both AST and ALT levels (Fig. 1a, b). The detailed meta-analysis results, including study selection, risk-of-bias assessment, and sensitivity analyses, are presented in Fig. S1, Tables S1 and S2. However, the moderate heterogeneity observed across studies in MASLD patients, as well as the potential role of gut microbiota as reported [14, 21], still warrants further investigation. To address this, we conducted a controlled intervention experiment in HFD-fed rats (Fig. 1c). Notably, Cur treatment significantly attenuated HFD-induced hepatic lipid accumulation compared with that in the HFD group (Fig. 1e-g). Additionally, Cur administration improved key metabolic parameters, including body weight, serum lipid profiles (triglycerides (TG), total cholesterol (TC), low-density lipoprotein cholesterol (LDL-C), and high-density lipoprotein cholesterol (HDL-C)), liver enzyme levels (ALT and AST), the liver index, and MASLD activity score (MAS) (Fig. 1d, i-p), confirming its efficacy in mitigating HFD-induced hepatic steatosis and injury. Strikingly, these beneficial effects were markedly attenuated in antibiotic-induced pseudo-germ-free rats (Fig. 1c-p). Measurements of Cur concentrations in plasma and colonic tissue further confirmed its low oral bioavailability and rapid metabolism following oral administration (Fig. S2), suggesting that Cur may ameliorate hepatic steatosis in a gut microbiota-dependent manner.

### Fecal microbiota transplantation from Cur-treated rats attenuated MASLD in HFD-fed rats

**Fig. 2:**
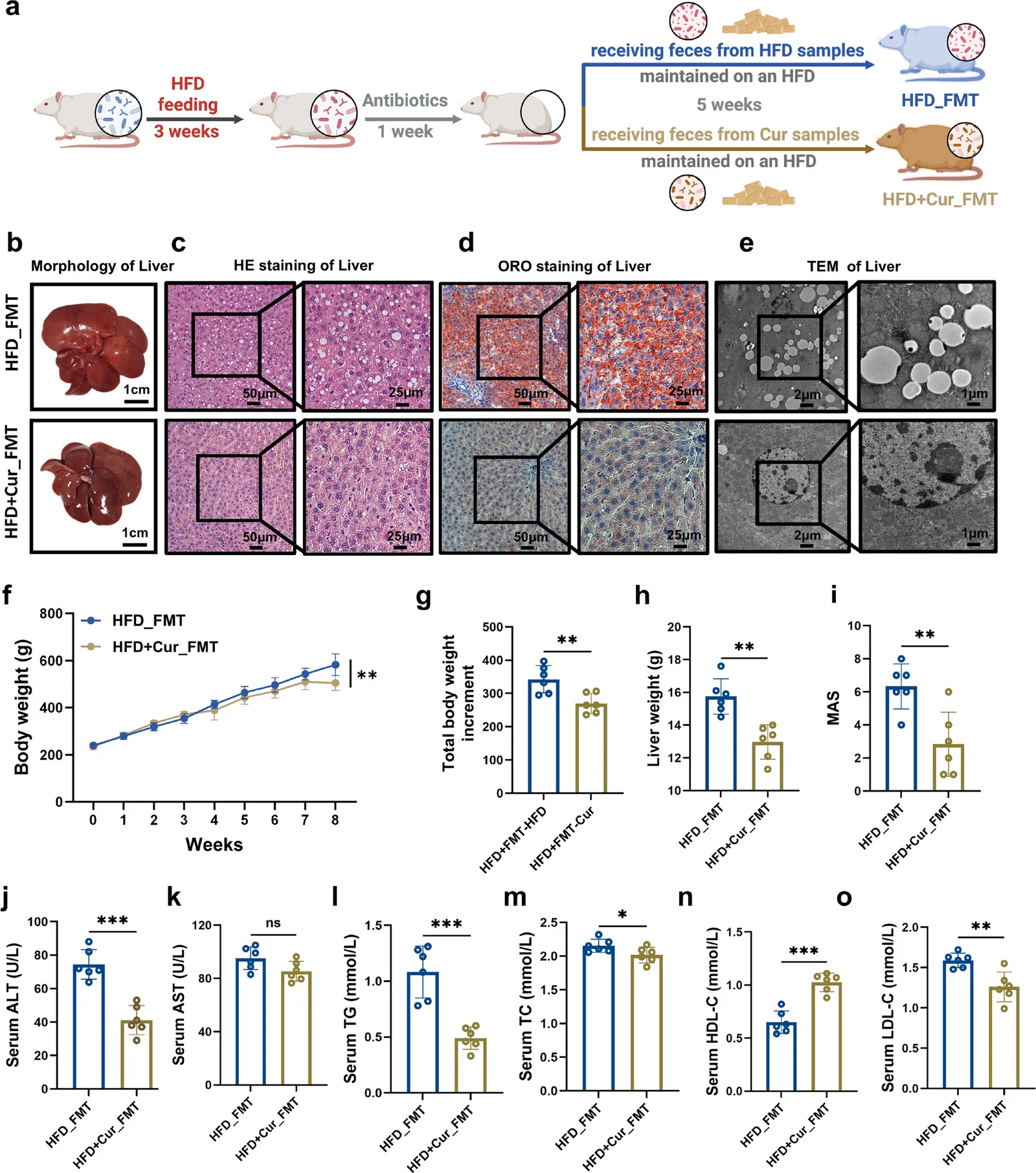
Fecal microbiota transplantation from Cur-treated rats ameliorated HFD-induced MASLD in rats. **a** Experimental design and workflow of the FMT study (*n* = 6). Created with BioRender. **b** Representative morphological images of livers from HFD + Cur_FMT and HFD_FMT rats (scale bar, 1 cm, *n* = 6). **c-d** H&E (**c**) and ORO (**d**) staining of liver tissues in the indicated groups (left scale bar, 50 μm and right scale bar, 25 μm, *n* = 6). **e** Transmission electron microscopy (TEM) images of hepatocytes in the HFD_FMT and HFD + Cur_FMT groups at 3600 × (left scale bar, 2 μm) and 8500 × (right scale bar, 1 μm) magnification (*n* = 6). **f** Dynamic body weight curve of recipient rats within the 8-week FMT intervention (*n* = 6). Baseline weight at Time 0 was measured post 1-week adaptation. **g** Quantification of total body weight increment after 8 weeks of FMT (*n* = 6). **h** Final liver weight of HFD + Cur_FMT and HFD_FMT rats harvested at experimental endpoint (*n* = 6). **i** MASLD activity score (MAS) in the indicated groups (*n* = 6). **j-o** Serum ALT (**j**), AST (**k**), TG (**l**), TC (**m**), HDL-C (**n**), and LDL-C (**o**) levels in the indicated groups (*n* = 6). All data are represented as the mean ± SD. *P* values were calculated by two-way ANOVA followed by Bonferroni post-test in (**f**), others with unpaired two-sided Student’s *t*-test. *P < 0.05, **P < 0.01, ***P < 0.001, ns: not significant

To evaluate whether the Cur-remodeled gut microbiota was sufficient to treat established MASLD, we employed fecal microbiota transplantation (FMT) in antibiotic-induced pseudo-germ-free rats (Fig. 2a). Recipients of microbiota from Cur-treated donors (HFD + Cur_FMT) displayed a marked attenuation of hepatic lipid deposition and steatosis compared with those receiving microbiota from HFD-fed donors (HFD_FMT) (Fig. 2b-d). Furthermore, the HFD + Cur_FMT group exhibited significant mitigation of mitochondrial damage and restoration of colonic villus integrity (Fig. 2e, Fig. S3, S4). Concomitantly, FMT from Cur-treated donors resulted in a pronounced decrease in body weight, body weight gain, liver weight, and MAS, as well as significant reductions in serum ALT, TG, TC, and LDL-C levels, while serum HDL-C levels were significantly increased (Fig. 2f-o). Collectively, these findings demonstrated that the therapeutic benefits of Cur against MASLD were dependent on gut microbiota modulation.

### Cur administration ameliorated HFD-induced gut microbiota dysbiosis and inhibited the growth of *C. sensu*

**Fig. 3:**
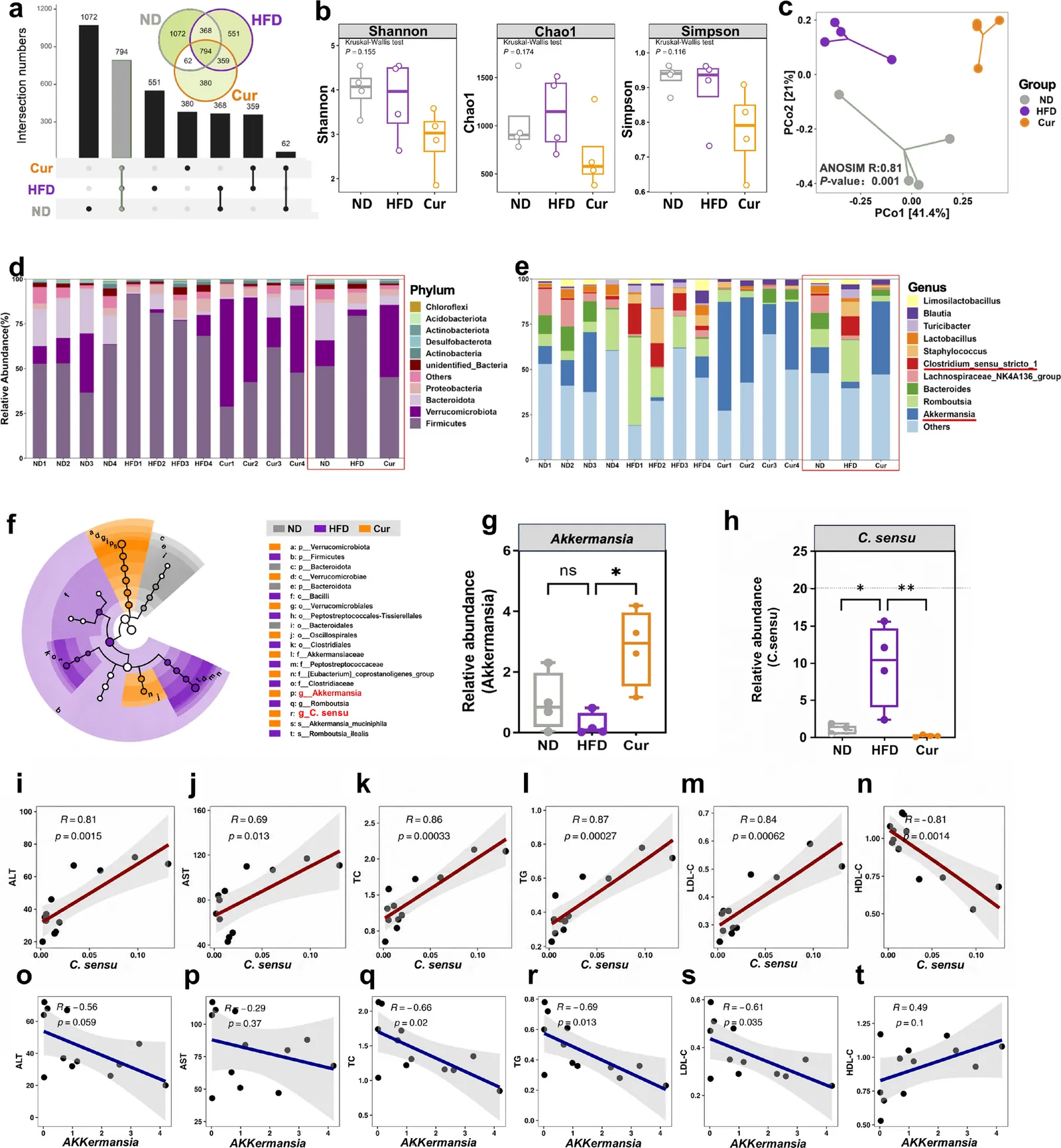
Cur administration ameliorated HFD-induced gut microbiota dysbiosis and specifically reduced the abundance of pathogenic *C. sensu*. **a** UpSet and Venn diagrams of the ASVs in ND, HFD, and Cur groups (*n* = 4). **b** Alpha diversity analysis using Shannon, Chao1, and Simpson indices at the ASV level (*n* = 4, K-W (Kruskal–Wallis) test). **c** PCoA using Bray–Curtis distance of beta diversity at the ASV level (*n* = 4, ANOSIM test). **d** Stacked bar plot showing the average relative abundance of top ten bacterial phyla. **e** Stacked bar plot showing the average relative abundance of top ten bacterial genera. **f** Cladograms generated by LEfSe depicting taxonomic association between microbiome communities from ND, HFD, and Cur rats. Grey, purple, and orange bars indicate taxa predominant in ND, HFD, and Cur rats, respectively. **g-h** The relative abundances of *Akkermansia* (**g**) and *C. sensu* (**h**) in the indicated groups (*n* = 4, one-way ANOVA followed by Tukey’s test). **i-n** Pearson correlation analysis between serum biochemical parameters and the relative abundance of *Clostridium_sensu_stricto_1*. Scatter plots showing the correlations of serum ALT (**i**), AST (**j**), TC (**k**), TG (**l**), LDL-C (**m**), and HDL-C (**n**) with the relative abundance of *Clostridium_sensu_stricto_1* (*n* = 4 rats per group; correlations were calculated across all 12 rats (*n*=12)). **o-t** Pearson correlation analysis between serum biochemical parameters and the relative abundance of *Akkermansia.* Scatter plots showing the correlations of serum ALT (**o**), AST (**p**), TC (**q**), TG (**r**), LDL-C (**s**), and HDL-C (**t**) with the relative abundance of *Akkermansia* (*n* = 4 per group; correlations were calculated across all 12 rats (*n*=12)). The red lines represent linear regression fits, and the shaded areas indicate 95% confidence intervals. Correlation coefficients R and *P-*values are displayed in the upper left corner of each panel. All data are represented as the mean ± SD. *P < 0.05, **P < 0.01, ***P < 0.001

**Fig. 4:**
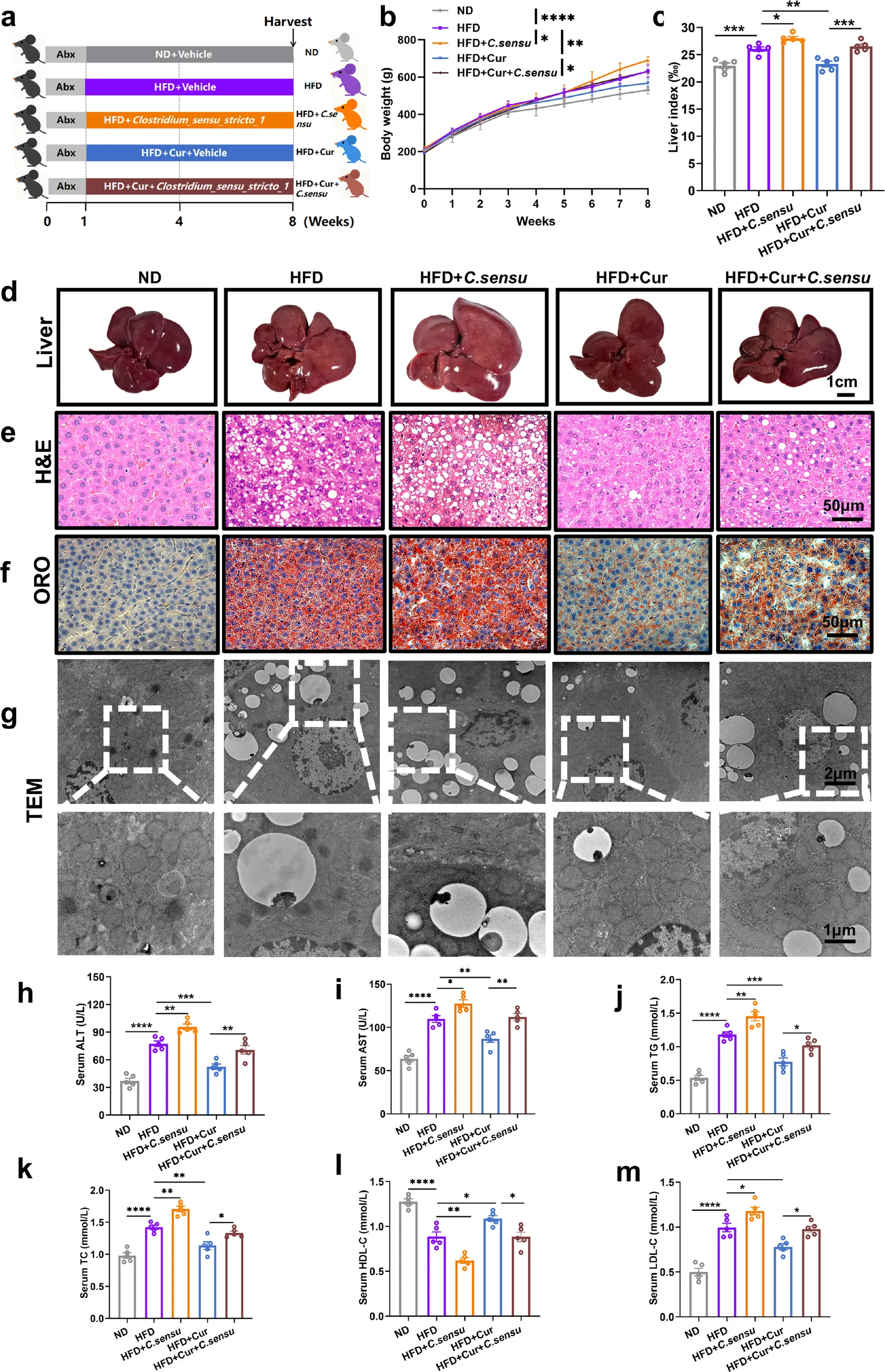
*C. sensu* abolished the ameliorative effects of Cur on MASLD. **a** Schematic diagram of the *C. sensu* administration rat model (*n* = 5). **b** Body weight of rats in ND, HFD, HFD + *C. sensu*, HFD + Cur, and HFD + Cur + *C. sensu* groups (*n* = 5). **c** Liver index for rats in the indicated groups (*n* = 5). **d** Representative morphological images of livers from the indicated groups (*n* = 5). **e–f** H&E and ORO staining of liver tissues in the indicated groups (scale bar, 50 μm, *n* = 5). **g** TEM images of hepatocytes in the indicated groups at 3600 × (upper scale bar, 2 μm) and 8500 × (lower scale bar, 1 μm) magnification (*n* = 5). **h-m** Serum ALT (**h**), AST (**i**), TG (**j**), TC (**k**), HDL-C (**l**), and LDL-C (**m**) levels in the indicated groups (*n* = 5). The abbreviation *C. sensu* represents *Clostridium_sensu_stricto_1*. All data are represented as the mean ± SD. *P* values were calculated by two-way ANOVA followed by Bonferroni post-test in (**b**), others with one-way ANOVA followed by Tukey’s test. *P < 0.05, **P < 0.01, ***P < 0.001, ****P < 0.0001

To elucidate the gut microbiome-mediated mechanisms underlying Cur’s therapeutic effects in an HFD-induced MASLD model, 16S rRNA sequencing of fecal samples derived from ND, HFD, and Cur rats was performed. The UpSet and Venn diagrams indicated that 794 shared ASVs were present in all groups (Fig. 3a). Alpha diversity indices, measured by different methodologies including Shannon, Chao1, Simpson, Goods_coverage, Pielou, ACE, and InvSimpson indices, demonstrated no significant differences in microbial diversity among the three indicated groups (Fig. 3b, Fig. S5a). Subsequent beta diversity analysis of PCoA and NMDS revealed distinct microbial compositions among rats in the ND, HFD, and Cur groups, further confirming the involvement of gut microbiota alteration in the process of HFD-induced MASLD pathogenesis and Cur-induced MASLD improvement (Fig. 3c, Fig. S5b). At the phylum and genus levels, Cur treatment partially modulated the gut microbial composition altered by HFD (Fig. 3d, e), and particularly reduced the abundance of the pathogenic bacterium *C. sensu* and increased that of the probiotic *Akkermansia* (Fig. 3f-h, Table S3). Among these, *C. sensu* exhibited a stronger correlation with key MASLD parameters than *Akkermansia* (Fig. 3i-t), consistent with previous reports implicating both *C. sensu* and *Akkermansia* in hepatic steatosis [22, 23]. The marked reduction of *C. sensu* and the induction of *Akkermansia* following Cur treatment suggested a pronounced effect, potentially contributing to Cur’s therapeutic efficacy against MASLD, with the Cur-induced reduction of *C. sensu* likely playing a more dominant role. To further investigate the role of *C. sensu* in Cur-mediated treatment of MASLD, antibiotic (Abx)-treated MASLD rats were subjected to targeted bacterial supplementation (Fig. 4a). Compared with HFD + vehicle rats, administration of *C. sensu* alone aggravated MASLD. Moreover, reintroduction of *C. sensu* significantly abolished the protective effects of Cur on MASLD, as evidenced by aggravated hepatic steatosis and deterioration of metabolic parameters (Fig. 4b-m). Collectively, these results provide evidence that *C. sensu* acted as a key pathogenic microorganism that counteracted Cur-mediated metabolic improvement, thereby highlighting its pivotal role in modulating the therapeutic response of MASLD to Cur.

### KYN, a key *Clostridium-*associated metabolite, mediated the protective effects of Cur in MASLD

To better understand the impact of Cur on the gut microbiota patterns, we conducted untargeted metabolomic profiling of liver tissues collected from HFD and Cur-treated rats. A total of 102 of 3,459 metabolites were identified as differentially abundant metabolites (DAMs), with a VIP score ≥ 2 based on PLS-DA analysis, including 50 upregulated and 52 downregulated compounds (Fig. S6a, b, Table S4). Functional enrichment analysis of differentially abundant metabolites was conducted with KEGG to better evaluate the functional alterations induced by Cur administration. Of interest, the DAMs were significantly enriched in pathways related to lipid metabolism and the regulation of lipolysis (Fig. S6c, d). Subsequently, we utilized the gutMGene database to predict 12 metabolites associated with *Clostridium* (Fig. S6e, Table S5) and integrated them with the 102 differentially abundant metabolites identified between the Cur and HFD groups, identifying kynurenine (KYN) as the core metabolite (Fig. S6f). Interestingly, previous studies have revealed that the gut microbiota converts dietary tryptophan into KYN via enzymes such as TDO2 and indoleamine 2,3-dioxygenase 1 (IDO1) [24], indicating that KYN, a key *Clostridium*-associated metabolite, mediates the protective effects of Cur. Moreover, we confirmed that Cur significantly reduced hepatic KYN levels, which showed positive correlations with ALT, TC, TG, and LDL-C (*P* < 0.01), and a negative correlation with HDL-C (*P* < 0.05) (Fig. S6g-m). This metabolic network was further validated by a global correlation heatmap (Fig. S6n), demonstrating a consistent pattern among *C. sensu*, KYN, and clinical metabolic parameters. To directly verify that *C. sensu is* a major contributor to KYN biosynthesis, we isolated *C. sensu* from the feces of HFD-fed rats by conducting large-scale characterizations of microbiota composition using 16S rRNA gene sequencing. We cultured *C. sensu* under anaerobic conditions for the indicated time and used liquid chromatography (LC)–MS technology to detect KYN levels in the supernatant. Indeed, *C. sensu* efficiently utilized tryptophan to produce high levels of KYN (Fig. S6o). Collectively, these findings identified KYN as a hepatotoxic metabolite derived from *C. sensu* in MASLD pathogenesis, and revealed that Cur exerted its therapeutic effects by specifically suppressing *C. sensu* and the subsequent production of KYN.

### Multi-dimensional integrative analysis identified CEBPB as a key target altered by genome-wide H3K27ac after Cur administration

**Fig. 5:**
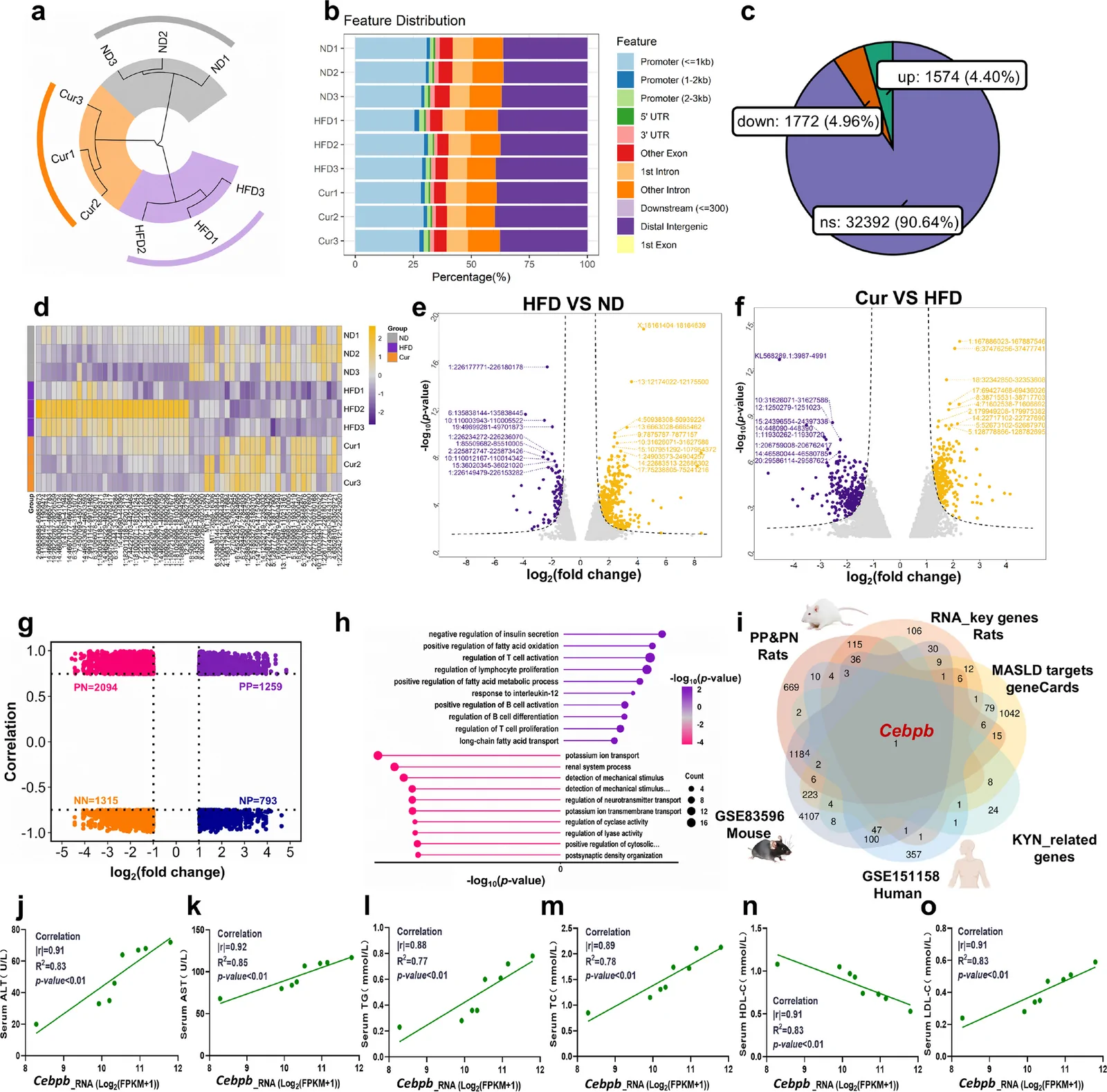
Cur supplementation robustly altered genome-wide H3K27ac profiles. **a** Sample distribution profiles of H3K27ac ChIP-seq data analyzed by hierarchical clustering (*n* = 3). **b** Distribution of H3K27ac peaks according to genomic regions in liver samples from the indicated groups (*n* = 3). **c** Pie chart showing the proportion of unchanged versus differentially acetylated H3K27ac peaks between livers from Cur and HFD groups (*n* = 3). **d** Heatmap highlighting the top 20 enriched H3K27ac peaks in liver samples from the ND, HFD, and Cur groups (*n* = 3). **e** Volcano plot indicating the differential H3K27ac peaks (orange, upregulated; purple, downregulated) between livers from HFD and ND groups (*n* = 3). **f** Volcano plot indicating the differential H3K27ac peaks (orange, upregulated; purple, downregulated) between livers from Cur and HFD groups (*n* = 3). **g** Genome-wide “four-way” plot showing the peak-genes generated by integrating analysis of ChIP-seq and RNA-seq between Cur and HFD groups. PP peak-genes (upregulated and positively correlated) are colored purple, NN peak-genes (downregulated and negatively correlated) are colored yellow, PN peak-genes (downregulated but positively correlated) are colored magenta, and NP peak-genes (upregulated but negatively correlated) are colored darkblue. **h** Pathway enrichment analysis of PP and PN peak-genes. Pathways enriched by PP peak-genes are colored purple and pathways enriched by PN peak-genes are colored magenta. **i** Venn diagram showing the overlapped genes between MASLD targets obtained from GeneCards® (https://www.genecards.org/), the PP and PN peak-genes, the RNA key genes, the KYN-related genes, GSE151158 and GSE83596. **j-o** Pearson correlation analysis of *Cebpb* expression from RNA-seq data and the MASLD phenotypic index (*n* = 4). All data are presented as mean ± SD. Correlation coefficients (R) and *P-*values are displayed in the upper left corner of each panel

Intriguingly, a preliminary study revealed that KYN can modulate key downstream genes by regulating H3K27ac (an important epigenetic modification that denotes active promoters and enhancers) [25]. To further validate these findings in this study, we investigated whether Cur exerts its effects via the functional consequences of the KYN pathway, and identified the critical genes affected by this epigenetic regulation. Integrated analysis of H3K27ac ChIP-seq and RNA-seq data was subsequently performed to identify differentially acetylated regions and their associated target genes across the ND, HFD, and Cur groups. As depicted in Fig. 5a, significant separations among the ND, HFD, and Cur groups were observed, confirming the reliability of the data. Notably, the majority of H3K27ac peaks were annotated as “Distal intergenic”, consistent with the property of enhancers that regulate gene expression from a distal location (Fig. 5b) [26]. Subsequently, differential analysis was performed to assess robustly modified peaks, and 3,346 differential peaks (including 1,574 upregulated and 1,772 downregulated peaks) were identified between the Cur and HFD groups (Fig. 5c). The volcano plot displayed the top distinct peaks between ND, HFD, and Cur groups, and a heatmap of differential peaks showed that Cur treatment partially restored histone modification patterns toward ND compared with HFD (Fig. 5d-f, Tables S6 and S7). Notably, these differential H3K27ac peaks following Cur treatment showed significant enrichment in lipid metabolism processes (Fig. S7a-h), suggesting their critical role in epigenetic reprogramming and the amelioration of hepatic steatosis in HFD-induced MASLD. Next, de novo motif analyses revealed that Cur-associated transcription factors (such as HNF1B, MATα2, NRF1, and NR2E3) are known to regulate metabolism, further confirming that Cur significantly altered the hepatic H3K27ac landscape to impact lipid catabolism processes (Fig. S7i-k, Tables S8-S10).

To further elucidate epigenetic regulation of gene expression by H3K27ac in Cur-triggered MASLD amelioration, we first performed RNA-seq and found that Cur treatment effectively reversed HFD-induced transcriptome alterations, particularly in genes involved in hepatic lipid homeostasis (Fig. S8, Tables S11-S13). Then we integrated these differentially expressed genes with H3K27ac alterations to further identify epigenetically regulated target genes. Notably, 1,259 PP peak-genes (Cor(peak-gene correlation) ≥ 0.75 and log_2_ fold change (RNA) ≥ 1) and 2,094 PN peak-genes (Cor(peak-gene correlation) ≥ 0.75 and log_2_ fold change (RNA) ≤ −1) were identified, highlighting H3K27ac’s crucial regulatory impact on gene expression (Fig. 5g, Table S14). Pathway enrichment analysis of PP and PN peak-genes revealed significant associations with MASLD-related pathways, aligning with the pathways identified from differential peaks of ChIP-seq and differentially expressed genes of RNA-seq data (Fig. 5h). Moreover, to identify core effector genes mediating Cur’s therapeutic effects in MASLD, we integrated multi-omics data including MASLD-related targets from GeneCards (https://www.genecards.org/), along with differentially acetylated peak-genes (PP and PN), transcriptomic key genes, and public datasets (GSE151158, GSE83596) (Fig. 5i). Among these, *Cebpb* emerged as a central gene showing strong positive correlations with MASLD phenotypic parameters (Fig. 5j-o); *it* is a well-known regulator of lipid synthesis and storage, involved in triglyceride accumulation and insulin resistance [27]. Additionally, by ChIP-seq and ChIP-qPCR analyses, we also found that H3K27ac density at the *Cebpb* enhancer (*chr3:156,625,975–156,626,736*) was increased in HFD rats compared with ND rats, which was effectively reversed by Cur treatment, highlighting the *Cebpb* enhancer (*chr3:156,625,975–156,626,736*) as an upstream epigenetic modulator of *Cebpb* expression reduction in Cur-triggered MASLD amelioration (Fig. S9a-e). To further validate the pathogenic role of *Cebpb* in MASLD, we analyzed three independent clinical datasets (GSE126484, GSE124868, GSE164084), all of which showed significantly elevated *CEBPB* expression in MASLD/MASH patients compared with healthy controls (Fig. S9f-h). Moreover, analysis of published single-cell RNA-sequencing data revealed hepatocyte-enriched *CEBPB* expression in MASLD, with *CEBPB* showing the highest expression in hepatocytes among major hepatic cell populations (Fig. S9i-l). Further quantitative analyses demonstrated significantly increased *CEBPB* expression and a higher proportion of *CEBPB*-positive hepatocytes in MASLD compared with normal controls (Fig. S9m-o). These findings supported hepatocyte-specific upregulation of *CEBPB* in MASLD and suggested that CEBPB might act as a key downstream effector of Cur-mediated anti-steatotic effects through regulation of hepatic lipid metabolism. Collectively, these data indicated that Cur supplementation remodeled the hepatic epigenetic landscape through H3K27ac modification, leading to targeted suppression of *Cebpb* expression and consequent amelioration of MASLD pathology.

### KYN binds to AHR to promote H3K27ac activation of *CEBPB*

To further investigate the connection between Cur-induced microbiota/metabolome dysbiosis and aberrant *CEBPB* expression mediated by epigenetic regulation, we performed transcription factor (TF) prediction for the *CEBPB* promoter and enhancer regions using the UCSC Genome Browser and the Transcription Regulation Database (GTRD). We identified 16 candidate TFs with potential regulatory activity toward *CEBPB*; subsequent protein–protein interaction (PPI) network analysis using the STRING database identified AHR as the most strongly associated TF (Fig. S10a). Cross-species sequence alignment using the UniProt database demonstrated strong evolutionary conservation of both *AHR* and *CEBPB* among humans, mice, and rats, supporting a conserved mechanism by which AHR may transcriptionally regulate *CEBPB* across species (Fig. S10b-d). Single-cell RNA-sequencing analysis further showed that *AHR* was enriched in hepatocytes among major hepatic cell populations during MASLD progression (Fig. S10e, f). Joint density visualization demonstrated the l co-expression of *AHR* and *CEBPB* within the hepatocyte compartment (Fig. S10g). In *AHR*⁺*CEBPB*⁺ hepatocytes, both *AHR* and *CEBPB* expression were significantly increased in MASLD compared with control cells (Fig. S10h). Moreover, single-cell correlation analysis revealed a positive association between *AHR* and *CEBPB* expression, with a stronger correlation observed in MASLD hepatocytes than in controls (Fig. S10i). These findings supported coordinated activation of *AHR* and *CEBPB* in diseased hepatocytes and provided single-cell evidence for the proposed AHR-*CEBPB* regulatory axis.

**Fig. 6:**
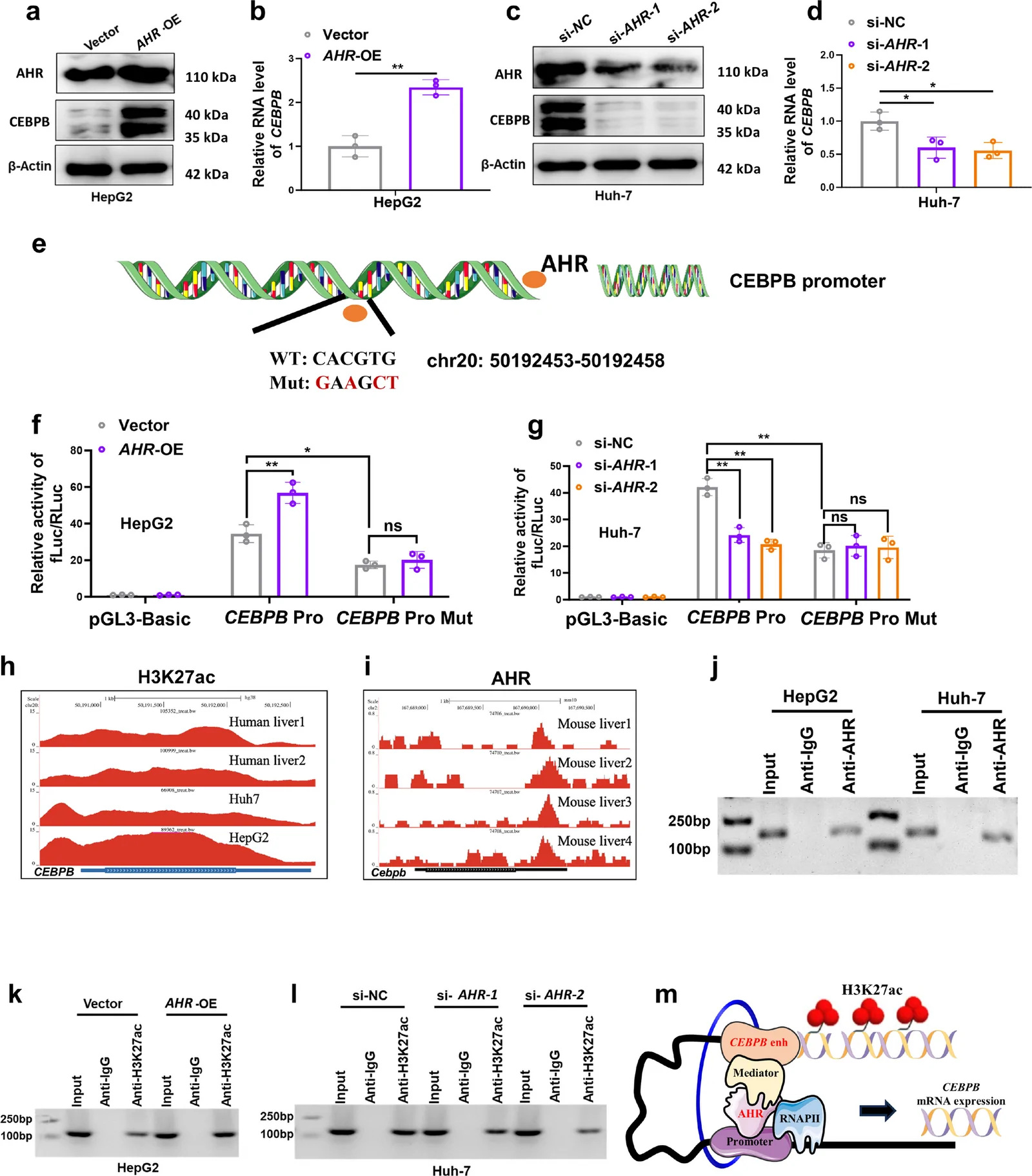
*CEBPB* is regulated by AHR-mediated H3K27ac activation. **a-b** CEBPB protein abundance (**a**) and corresponding mRNA levels (**b**) in OA (0.6 mM)-induced HepG2 cells transfected with *AHR* overexpression plasmid (*AHR*-OE) or empty vector (Vector) were examined by Western blotting and qRT-PCR, respectively (*n* = 3). **c-d** The protein (**c**) and mRNA (**d**) levels of *CEBPB* in OA (0.6 mM)-induced Huh-7 cells transfected with siRNAs against *AHR* (si-*AHR*−1 and si-*AHR*−2) or negative control siRNA (si-NC) were determined by Western blotting and qRT-PCR, respectively (*n* = 3). **e** A schematic diagram illustrating the predicted wild-type and mutant AHR-binding sites in the *CEBPB* promoter. **f-g** The transcriptional activity of wild-type and mutant *CEBPB* promoter constructs in response to AHR was determined by luciferase reporter assay, with Renilla luciferase activity serving as an input control (*n* = 3). **h** ChIP-seq data from the Cistrome database (accession numbers: GSM1670897, GSM2360939) show the enrichment of H3K27ac around the promoter region of *CEBPB* in human liver tissues, HepG2, and Huh-7 cells. **i** ChIP-seq data from the Cistrome database (accession numbers: ENCSR382MOM_1, ENCSR994YLZ_2, ENCSR994YLZ_1) reveal the enrichment of AHR around the promoter region of *Cebpb* in mouse liver tissues. **j** ChIP-qPCR validation of specific AHR occupancy at the *Cebpb* promoter region (*n* = 3/group, two-way ANOVA). ChIP was conducted with AHR antibody and non-specific IgG control. **k-l** ChIP assays were performed to determine whether AHR modulates H3K27ac enrichment at the *CEBPB* regulatory locus in HepG2 (**k**) and Huh-7 (**l**) cells. **m** A schematic diagram depicting an interactive model that AHR transcriptionally activates *CEBPB* expression. Results were presented as mean ± SD. *P* values were calculated with unpaired two-sided Student’s *t*-test in (**b**) and** f-g**, one-way ANOVA followed by Tukey’s test in **d**. *P < 0.05, **P < 0.01, ***P < 0.001, ns: not significant

Given the established role of the KYN/AHR pathway in various pathologies [12, 28, 29], we investigated whether KYN drives *CEBPB* transcription through AHR signaling. We first confirmed AHR as a transcriptional regulator of *CEBPB*. In oleic acid (OA)-treated hepatocytes, *AHR* overexpression in HepG2 cells markedly increased *CEBPB* mRNA and protein levels, whereas *AHR* silencing in Huh-7 cells yielded the opposite effects (Fig. 6a-d). Furthermore, we cloned a 2-kb wild-type or AHR*-*binding-site-mutated *CEBPB* promoter into a pGL3 reporter vector (Fig. 6e). Luciferase reporter assays confirmed that AHR strongly transactivated the wild-type promoter, whereas mutagenesis of AHR-binding motifs completely abolished this induction (Fig. 6f, g). Additionally, the potential co-regulation of *CEBPB* by AHR and H3K27ac was supported by Cistrome data, which revealed substantial H3K27ac enrichment at the *CEBPB* locus in human liver cells and prominent AHR binding peaks in mouse liver tissue (Fig. 6h, i). To further validate the direct interaction between AHR and the regulatory region of *CEBPB*, ChIP-qPCR analysis demonstrated a significant enrichment of AHR at the predicted *CEBPB* promoter compared with the negative control, confirming the direct occupancy of AHR at the *CEBPB* regulatory locus (Fig. 6j). Consistently, ChIP assays revealed that *AHR* overexpression markedly increased H3K27ac enrichment at the *CEBPB* locus in HepG2 cells (Fig. 6k), whereas *AHR* depletion significantly reduced H3K27ac enrichment in Huh-7 cells (Fig. 6l). These findings underscored the critical role of AHR in promoting *CEBPB* transcriptional activation by enhancing H3K27ac modification (Fig. 6m).

**Fig. 7:**
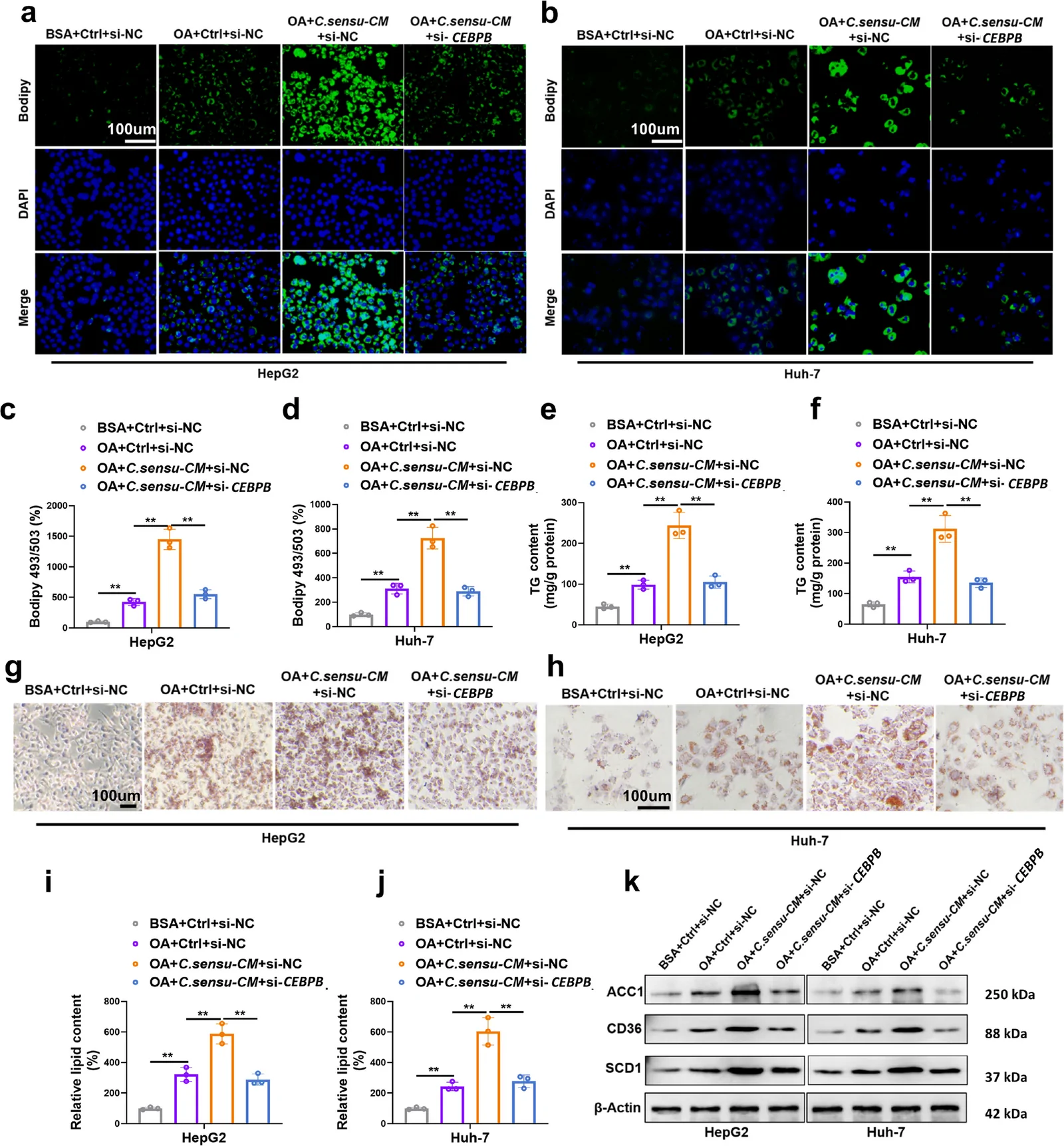
*C. sensu* regulated lipid accumulation in hepatocytes in a CEBPB-dependent manner. **a-d** Representative BODIPY 493/503 fluorescence staining of lipid droplet accumulation and quantitative analysis in HepG2 and Huh-7 cells treated with *C. sensu* or control and transfected with siRNAs against *CEBPB* (si-*CEBPB*) or negative control siRNA (si-NC) with or without OA (0.6 mM) treatment (scale bar, 100 μm, *n* = 3). **e–f** Quantification of intracellular TG content in HepG2 and Huh-7 cells treated with *C. sensu* or control transfected with si-*CEBPB* or si-NC with or without OA treatment (0.6 mM, *n* = 3). **g-j** Representative ORO staining of lipid droplet accumulation and quantitative analysis in HepG2 and Huh-7 cells treated with *C. sensu* or Control and transfected with si-*CEBPB* or si-NC, with or without OA (0.6 mM) treatment (scale bar, 100 μm, *n* = 3). **k** The levels of lipid transport- and synthesis-related proteins (*ACC1*, *CD36*, and *SCD1*) in *C. sensu*- or control-treated HepG2 and Huh-7 cells transfected with si-*CEBPB* or si-NC with or without OA treatment (0.6 mM) were assessed by Western blotting (*n* = 3). Results were presented as mean ± SD from three independent experiments. *P* values were calculated with one-way ANOVA followed by Tukey’s test. The abbreviation *C. sensu* represents *Clostridium_sensu_stricto_1*. *P < 0.05, **P < 0.01, ***P < 0.001

Subsequently, we investigated the critical role of CEBPB activation in mediating lipid deposition induced by *C. sensu* or its metabolite KYN. We found that supplementation with *C. sensu* supernatants significantly exacerbated OA-induced lipid deposition and intracellular TG accumulation in HepG2 and Huh-7 cells, as evidenced by BODIPY 493/503 and Oil Red O staining. Additionally, the supernatants upregulated lipid transport and synthesis genes (*ACC1*, *CD36*, and *SCD1*). Crucially, these pro-steatotic effects were effectively reversed by *CEBPB* depletion (Fig. 7a-k). Notably, replacing the supernatants with commercial KYN recapitulated these phenotypes. KYN treatment similarly aggravated lipid accumulation and upregulated the expression of lipid metabolism genes, which were also mitigated by *CEBPB* knockdown (Fig. S11).

These results demonstrated that *C. sensu*-derived KYN promoted hepatic steatosis through AHR-mediated *CEBPB* activation and subsequent modulation of lipid metabolic pathways.

### CEBPB activated the PI3K-AKT pathway to promote hepatic steatosis

To characterize the global regulatory function of *CEBPB* in hepatic steatosis, we performed RNA-seq in Huh-7 cells transfected with *CEBPB-targeting* siRNA or negative control siRNA. Principal component analysis (PCA) showed distinct clustering of the two groups, confirming the reliability and reproducibility of the sequencing data (Fig. S12a). Using thresholds of |log_2_(fold change)|≥ 1 and *P* < 0.05, we identified 438 differentially expressed genes (DEGs), including 343 downregulated and 95 upregulated genes (Fig. S12b, c). Functional enrichment analysis revealed that these genes were significantly involved in MASLD-related processes, such as the “fatty acid metabolic process” (Fig. S12d). KEGG pathway analysis further revealed that the PI3K-AKT signaling pathway was the most significantly enriched pathway (Fig. S12e, f), which is a key pathway regulating lipid metabolism and inflammation in MASLD [30–32]. These findings were functionally validated in OA-treated HepG2 and Huh-7 cells, where KYN-induced increases in CEBPB, p-PI3K, and p-AKT protein levels were markedly attenuated upon *CEBPB* depletion (Fig. S12g). Collectively, these findings established that KYN contributed to hepatic steatosis by activating the AHR/CEBPB/PI3K/AKT signaling axis.

### Reduced hepatic KYN levels mediated the protective effects of Cur in MASLD

**Fig. 8:**
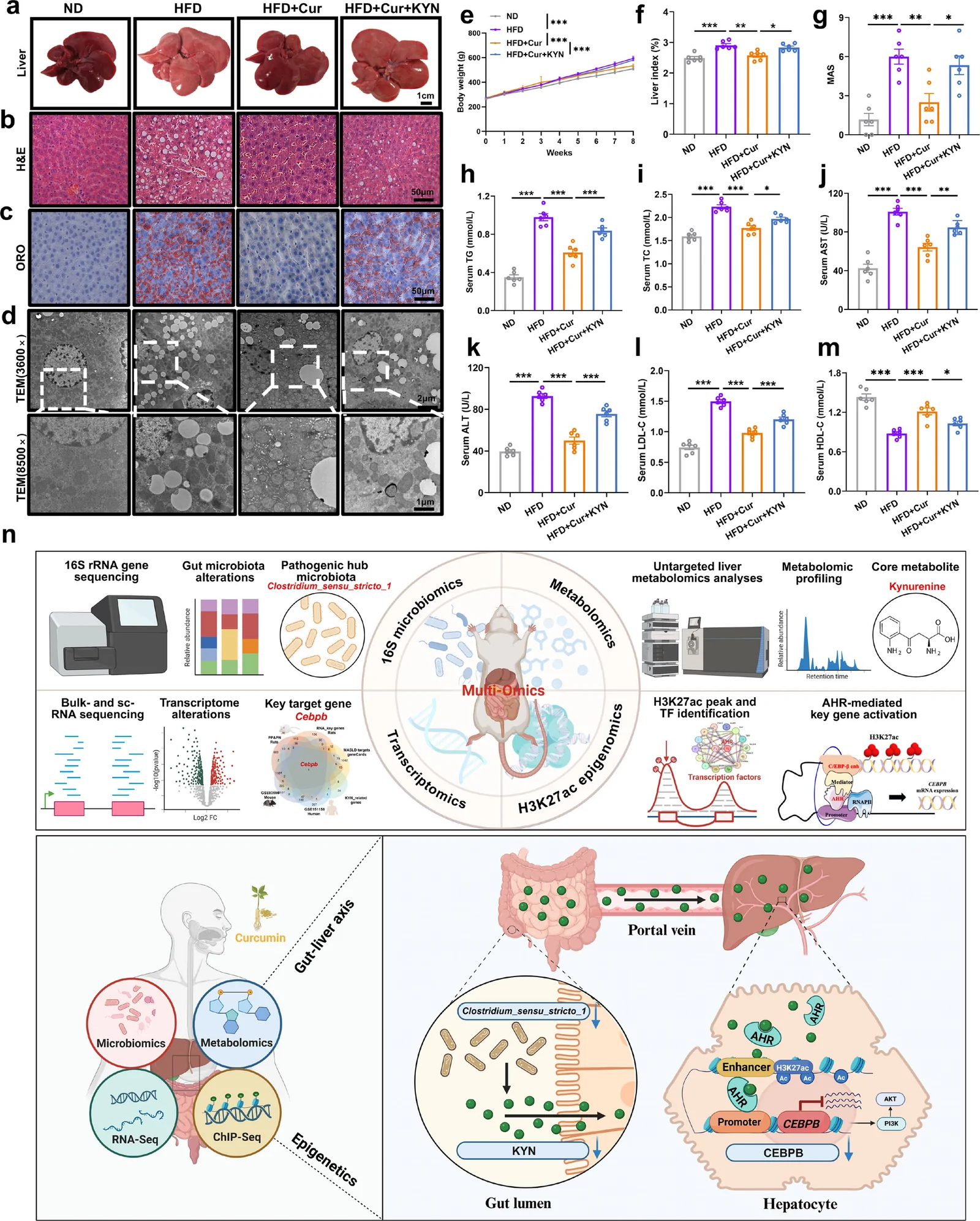
Reduced KYN plays a protective role in Cur-regulated lipid steatosis in MASLD. **a** Representative morphology images of livers from rats fed ND, HFD, HFD + Cur, and HFD + Cur + KYN (scale bar, 1 cm, *n* = 6). **b** Histopathological section of liver tissue by hematoxylin and eosin staining (scale bar, 50 μm, *n* = 6). **c** Oil Red O (ORO) staining of the livers from the indicated groups (scale bar, 50 μm, *n* = 6). **d** Transmission electron microscopy (TEM) of hepatocytes in ND, HFD, HFD + Cur, and HFD + Cur + KYN groups at 3600 × (upper, scale bar, 2 μm) and 8500 × (lower, scale bar, 1 μm) magnification (*n* = 6). **e** Body weight of ND, HFD, HFD + Cur, and HFD + Cur + KYN rats (*n* = 6). **f** Liver index (ratio of liver weight to body weight) was calculated for ND, HFD, HFD + Cur, and HFD + Cur + KYN rats (*n* = 6). **g** MASLD activity score (MAS) in the indicated groups (*n* = 6). **h-m** Serum TG (**h**), TC (**i**), AST (**j**), ALT (**k**), LDL-C (**l**), and HDL-C (**m**) levels in the indicated groups (*n* = 6). **n**. Graphical abstract. A comprehensive multi-omics study integrating 16S microbiomics, metabolomics, transcriptomics, and H3K27ac epigenomics was conducted to elucidate the mechanisms of Cur’s anti-MASLD effect. Oral administration of Cur demonstrated a beneficial impact on MASLD by modulating the gut microbiota, notably suppressing the pathogenic bacterium *C. sensu*, which subsequently led to a decrease in KYN accumulation. Mechanistically, gut microbiota-derived KYN binds to AHR, recruiting the epigenetic histone modification H3K27ac to specifically regulate the key gene *Cebpb*, further activating PI3K-AKT pathway to aggravate hepatic steatosis. Collectively, this study reveals a novel gut-liver axis in MASLD pathogenesis, demonstrating that Cur alleviates hepatic steatosis by targeting the microbiota-KYN-AHR-H3K27ac-*Cebpb* epigenetic axis, thereby positioning both Cur and KYN modulation as promising therapeutic strategies for MASLD intervention (Created with BioRender). All data are represented as the mean ± SD. *P* values were calculated by two-way ANOVA followed by Bonferroni post-test in (**e**), others with one-way ANOVA followed by Tukey’s test. *P < 0.05, **P < 0.01, ***P < 0.001. ALT: alanine aminotransferase, AST: aspartate aminotransferase, TG: triglycerides, TC: total cholesterol, HDL-C: high-density lipoprotein cholesterol, LDL-C: low-density lipoprotein cholesterol

Building upon our previous discovery that KYN acts as a critical metabolic disruptor through AHR-mediated epigenetic reprogramming of hepatic lipid metabolism [33], we sought to investigate its role in modulating Cur’s anti-steatotic effects by using a well-controlled intervention study in HFD-fed rats. Consistently, Cur treatment significantly ameliorated hepatic lipid accumulation compared with the HFD group, while KYN co-administration largely abolished these protective effects (Fig. 8a-e). Moreover, Cur-mediated improvements in metabolic parameters were significantly compromised by KYN, including liver index, MAS score, serum TG, TC, ALT, AST, LDL-C, and HDL-C (Fig. 8f-m). To further explore the underlying mechanism, we performed AHR-CEBPB co-localization analysis. As shown in Fig. S13, substantial spatial co-expression of AHR and CEBPB was observed in liver tissues, supporting the involvement of the AHR-CEBPB signaling axis in KYN-mediated antagonism of Cur’s protective effects. Therefore, these findings not only confirmed KYN’s pathogenic role in promoting hepatic lipid accumulation but also demonstrated its ability to functionally counteract Cur’s therapeutic effects.

## Discussion

The escalating global prevalence of MASLD amidst the obesity pandemic underscores the urgent need for novel therapeutic strategies [34, 35]. By integrating multi-omics data, including 16S microbiomics, metabolomics, H3K27ac epigenomics, bulk transcriptomics, and single-cell transcriptomics, our study provides a comprehensive understanding of the mechanisms underlying the therapeutic effects of Cur on HFD-induced MASLD. Briefly, Cur ameliorates hepatic steatosis and metabolic dysfunction in a gut microbiota-dependent manner by suppressing the pathogenic genus *C. sensu*. This reduces hepatic KYN-mediated activation of AHR, decreases H3K27ac enrichment at the *Cebpb* locus, suppresses PI3K-AKT signaling, and ultimately alleviates hepatic steatosis. Overall, this study uncovers a novel gut-liver axis in MASLD pathogenesis, demonstrating a mechanistic link between the gut microbiota-derived metabolite (KYN), epigenetic reprogramming (AHR/H3K27ac), and transcriptional regulation of lipid metabolism (*Cebpb*) (Fig. 8n).

Cur, a bioactive polyphenol from traditional Chinese medicine, exhibits diverse pharmacological properties, including antioxidant, anti-inflammatory, and metabolic regulatory effects [36–39], and has potential therapeutic effects against a wide range of diseases, including MASLD [40, 41]. Considerable efforts have been devoted to identifying the molecular targets through which Cur exerts its therapeutic effects against MASLD, revealing several candidate targets, such as LXRα, PI3K, HIF-1, HMGB1, NF-κB, TIMP-1, and 8-OHdG [32, 42–44]. Notably, these studies have primarily attributed the observed effects to the direct action of Cur. Considering its extremely low bioavailability and high intestinal enrichment, researchers have gradually begun to recognize the possibility that Cur improves MASLD via the gut microbiota [45–47]. He et al*.*, for example, demonstrated in a clinical trial that improvements in gut microbiota-dependent bile acid (BA) metabolism after Cur intervention were associated with a reduction in hepatic fat content in patients with MASLD [14]. However, their study remained at the correlational level. In this study, we further confirmed the critical role of the gut microbiota in Cur-mediated improvement of MASLD through antibiotic treatment and FMT experiments and identified *Clostridium sensu stricto 1* (*C. sensu*) as a potential microbial contributor, consistent with previous reports linking this genus to inflammation, metabolic disorders, and intestinal mucosal damage [48, 49]. Wen et al*.* further linked *C. sensu* to intestinal inflammation, supporting its potential involvement in intestinal barrier dysfunction [50]. However, Hahn et al*.* revealed that *C. sensu* abundance significantly decreases with the onset of hepatic fat accumulation and progression of fibrosis, suggesting a protective role of *the* genus against MASLD [51–53]. Therefore, the intrageneric complexity of *C. sensu* and its species-dependent effects in the complex in vivo environment warrant further investigation.

KYN, a major metabolite generated from tryptophan catabolism via the kynurenine pathway, has been increasingly recognized for its roles in various liver diseases, particularly MASLD [54, 55]. It has been reported that the interaction between the gut microbiome and the KYN pathway has emerged as a significant area of research, particularly in the context of metabolic diseases and immune regulation [56, 57]. Sun et al*.* revealed that the gut microbiota metabolizes dietary tryptophan into KYN through enzymatic conversion by TDO2 and IDO1, and that *C. sensu* has been specifically implicated in driving elevated KYN levels in MASLD [56]. Dietary components that influence gut microbiota composition, such as prebiotics and polyphenols, regulate KYN pathway activity, highlighting the therapeutic potential of targeting this pathway [58]. Consistent with these findings, our preliminary studies also revealed that H3K27ac/TDO2-induced KYN was a critical driver of macrophage M1 polarization and MASLD progression [12]. Mechanistically, Mezrich et al. elucidated that KYN serves as an endogenous ligand for AHR, inducing anti-inflammatory effects by promoting the differentiation of regulatory T cells (Tregs) and dampening pro-inflammatory pathways [59]. Importantly, although KYN was identified as a key mediator linking gut microbiota alterations to hepatic epigenetic remodeling in our study, it should not be considered the sole determinant of Cur-mediated therapeutic effects. Cur-induced metabolic improvement likely involves a broader network of microbial-derived metabolites and community-level microbiota remodeling. Other altered metabolites, including bile acids, short-chain fatty acids, and additional tryptophan-derived metabolites, may contribute to the beneficial effects of Cur through complementary mechanisms. Therefore, the biological consequences of KYN signaling are determined by the integrated effects of the tissue-specific microenvironment, cellular context, disease state, local metabolite availability, and downstream signaling pathways.

Although this study elucidates a novel mechanistic paradigm for Cur’s therapeutic effects in MASLD, delineating a coordinated gut-liver axis involving microbial-metabolic-epigenetic crosstalk, several limitations should be acknowledged. In particular, direct in vivo causal validation of this axis will require future genetic models. Moreover, several translational and biological considerations warrant further investigation. First, significant differences in gut microbiota composition and metabolite profiles between rodents and humans present a major translational challenge; the pathogenic role of *C. sensu* and its KYN metabolism may not be directly recapitulated in human MASLD, necessitating validation using humanized gnotobiotic models and clinical cohorts. Given that Cur induces remodeling of multiple gut microbiota populations, it remains to be further investigated whether its metabolic therapeutic effects are mediated solely through the suppression of pathogenic bacteria or also involve the upregulation of beneficial bacterial communities. Second, oral Cur is known to exert pleiotropic effects, including direct antioxidant, anti-inflammatory, and iron-chelating activities, raising the possibility of off-target actions independent of the microbiota–epigenetic axis. Therefore, genetic or pharmacological ablation of the pathway components (e.g., AHR or CEBPB) in hepatocyte-specific models would be required to delineate target-specific contributions. Third, although HepG2 and Huh-7 cells are widely used models for investigating hepatic lipid metabolism, they cannot fully recapitulate the biological characteristics of normal hepatocytes. Future studies using primary hepatocytes or non-transformed hepatocyte models will be valuable to further validate the mechanisms identified in the present study. Furthermore, although supportive single-cell data suggest enrichment of H3K27ac–*Cebpb* signaling in hepatocytes, the current resolution cannot exclude contributions from non-parenchymal cells such as Kupffer cells or hepatic stellate cells, which also express AHR and may modulate steatosis indirectly. Future single-cell multi-omics analyses (e.g., ATAC-seq combined with RNA-seq) are needed to confirm hepatocyte-specific epigenetic reprogramming. Finally, our 8-week high-fat diet model primarily captures early steatosis without substantial fibrosis or carcinogenesis. Whether chronic Cur treatment or KYN reduction can prevent progression to metabolic dysfunction-associated steatohepatitis (MASH), fibrosis, or hepatocellular carcinoma remains unknown, and long-term studies with extended dietary challenges or genetic susceptibility models (e.g., *Pten* or MUP-uPA mice) are required to assess the durability and safety of targeting this axis.

Overall, this study supports a gut microbiota-metabolite-epigenetic pathway through which Cur ameliorates experimental MASLD. Specifically, Cur suppressed *C. sensu*, reduced hepatic KYN accumulation, and consequently attenuated AHR activation and H3K27ac enrichment at the *Cebpb* locus, leading to decreased *Cebpb* expression, reduced PI3K-AKT signaling, and diminished hepatic lipid accumulation. This mechanistic framework advances our understanding of Cur’s therapeutic actions, identifies KYN, AHR, and CEBPB as candidate targets for MASLD intervention, and highlights the therapeutic potential of modulating the gut-liver axis.

## Materials and methods

### Animals and treatment

Male Sprague–Dawley rats (6 weeks old) were obtained from Beijing Vital River Laboratory Animal Technology Co., Ltd. (Beijing, China) and housed under specific pathogen-free (SPF) conditions. After 7 days of acclimatization, rats were randomly assigned to receive either a matched normal diet (XTCON50H, Xietong, China; protein 20.10%, fat 10.00%, and carbohydrate 69.90% of total energy) or a high-fat diet (XTHF45, Xietong, China; protein 20.10%, fat 45.10%, and carbohydrate 34.70% of total energy) for 8 weeks to establish the MASLD model(Table S15). Body weight was recorded weekly throughout the intervention.

Beginning in week 4, HFD-fed rats were randomly assigned to the HFD, Cur, and Cur + Abx groups to evaluate the protective effects of Cur against hepatic steatosis, and the assigned treatments continued through week 8. Rats in the HFD and Cur groups received sodium carboxymethyl cellulose (CMC-Na) or Cur (80 mg/kg/day in CMC-Na), respectively, by oral gavage every other day for 5 consecutive weeks. The Cur + Abx group received the same Cur regimen together with broad-spectrum antibiotics in drinking water (vancomycin, 0.5 g/L; neomycin, 1 g/L; metronidazole, 1 g/L; and ampicillin, 1 g/L). ND rats received CMC-Na as the control. After 8 weeks, rats were fasted for 12 h before euthanasia. Serum, tissue, and fecal samples were collected and stored at −80 °C for further analyses.

To investigate the role of KYN in modulating the anti-steatotic effects of Cur, rats were divided into four groups: ND, HFD, HFD + Cur, and HFD + Cur + KYN. Beginning at week 4 and continuing until the end of week 8, rats in the HFD + Cur and HFD + Cur + KYN groups received Cur (80 mg/kg) by oral gavage every other day, while rats in the HFD + Cur + KYN group additionally received KYN (30 mg/kg) [60, 61]. Rats in the ND and HFD groups received an equivalent volume of CMC-Na vehicle. ND rats received CMC-Na as the control. At the end of week 8, rats were fasted for 12 h and euthanized. Blood, tissue, and fecal samples were collected and stored at −80 °C.

The fecal microbiota transplantation (FMT) procedure was performed as illustrated in Fig. 2a [62, 63]. Colonic fecal samples were collected from HFD-fed and Cur-treated donor rats. Fecal pellets were homogenized in phosphate-buffered saline containing 20% glycerol at a concentration of 20 mg/mL; after centrifugation at 100 × g for 10 min, the supernatants were aliquoted and stored at −80 °C until transplantation. Twelve recipient rats underwent 1 week of acclimatization, 3 weeks of HFD pre-induction, and 1 week of broad-spectrum antibiotic treatment in drinking water. After microbiota depletion, rats were randomly assigned to two groups: the HFD + Cur_FMT group received fecal suspensions from Cur-treated donors by oral gavage every other day, whereas the HFD_FMT group received fecal suspensions from HFD-fed donors. FMT was continued for 5 weeks with HFD feeding throughout the intervention. Body weight was recorded weekly. At the endpoint, rats were euthanized, and serum, tissue, and fecal samples were collected and stored at −80 °C for subsequent analyses.

For the *C. sensu* supplementation rescue assay, all rats underwent 1 week of antibiotic pretreatment to deplete the intestinal microbiota and were then randomly assigned to five groups: (1) ND + vehicle; (2) HFD + vehicle; (3) HFD + *C. sensu*; (4) HFD + Cur + vehicle; and (5) HFD + Cur + *C. sensu*. All HFD groups received the HFD continuously for 8 weeks: 3 weeks for model establishment, followed by 5 weeks of continued HFD feeding and daily oral gavage. At the endpoint, rats were euthanized according to institutional animal ethics protocols, and blood, tissue, and fecal samples were collected and stored at −80 °C for subsequent analyses.

### Histological and biochemical analyses

MAS was scored according to Kleiner et al. [64]. For ultrastructural analysis, liver specimens were fixed with glutaraldehyde and osmium tetroxide and examined by transmission electron microscopy using a Talos L120C G2 microscope (Thermo Fisher Scientific, USA). Serum levels of alanine aminotransferase (ALT), aspartate aminotransferase (AST), total cholesterol (TC), triglycerides (TG), high-density lipoprotein cholesterol (HDL-C), and low-density lipoprotein cholesterol (LDL-C) were measured using a Hitachi 3100 automatic biochemical analyzer according to the manufacturer’s instructions [12].

### 16S rRNA gene sequencing

Microbial DNA was extracted from rat fecal samples, and 16S rRNA gene sequencing was performed using an Illumina MiSeq platform. After quality filtering, amplicon sequence variants (ASVs) were generated using QIIME2 (v2022.2) and taxonomically annotated against the SILVA database [65, 66]. Microbial α- and β-diversity analyses were performed using the R package vegan [67]. LEfSe was used to identify differentially abundant taxa [68], and PICRUSt was used to predict the functional profiles of microbial communities [69].

### Untargeted liver metabolomics analysis

Untargeted metabolomic profiling of liver samples was performed using an LC–MS/MS platform (UHPLC coupled with a Q Exactive HFX Orbitrap mass spectrometer; Thermo Fisher Scientific) as previously described [70].

### H3K27ac ChIP-seq

H3K27ac ChIP-seq was performed using a modified version of an established protocol [71]. Briefly, approximately 150 mg of liver tissue was cross-linked, fragmented, and immunoprecipitated with an anti-H3K27ac antibody. Purified DNA was used for library construction and sequencing. Clean reads were aligned to the rat genome (Rattus_norvegicus_6.0) using BWA, and peaks were identified using MACS2 (q < 1 × 10⁻^5^). Differential H3K27ac peaks were identified using DESeq2 with |log_2_(fold change)|≥ 1 and *P* < 0.05 [72]. Motif enrichment was analyzed using HOMER, and genomic signals were visualized using IGV.

### RNA-seq and integrative multi-omics analysis

RNA-seq was performed as previously described [71], and differentially expressed genes were identified using DESeq2 with |log_2_(fold change)|≥ 1 and *P* < 0.05 [72]. H3K27ac ChIP-seq and RNA-seq datasets were integrated to identify coordinated epigenetic and transcriptional alterations as previously reported [73, 74]. Peak–gene with a Pearson correlation coefficient ≥ 0.75 and |log_2_(fold change)|≥ 1 were retained for subsequent analyses.

### RT-qPCR and luciferase reporter assay

Total RNA isolated from liver tissues and cells was reverse-transcribed into cDNA, which was then subjected to SYBR Green-based qPCR. Primer sequences are provided in Table S16. For luciferase assays, the 2-kb *CEBPB* promoter and its site-directed mutants were cloned into the pGL3-Basic vector. The pRL-TK plasmid was cotransfected as an internal control, and luciferase activity was measured as previously described [75].

### Functional enrichment analysis

Gene Ontology (GO) and Kyoto Encyclopedia of Genes and Genomes (KEGG) enrichment analyses, together with gene set enrichment analysis (GSEA), were performed using the R package clusterProfiler (v4.11.1) [76]. *P* < 0.05 was considered statistically significant.

### Cell culture, transfection, and lipid accumulation assays

Huh-7 (RRID: CVCL_0336) and HepG2 (RRID: CVCL_0027) cells were obtained from the Type Culture Collection of the Chinese Academy of Sciences and routinely confirmed to be mycoplasma-free. Cells were cultured in DMEM supplemented with 10% fetal bovine serum and 1% penicillin–streptomycin. siRNAs targeting *AHR* and *CEBPB* and an *AHR* overexpression plasmid were transfected into cells using Lipofectamine 2000. Oligonucleotide sequences are provided in Table S17.

Intracellular lipid accumulation was induced in cultured cells using 0.6 mM oleic acid (OA). Intracellular lipid deposition was assessed by Oil Red O and BODIPY 493/503 staining [77]. Intracellular TG levels were measured using a commercial kit and normalized to total protein concentrations determined using a BCA assay [12].

### Western blot

Western blotting was performed as previously described [78]. The primary antibodies used were as follows: AHR (67785–1-Ig, Proteintech, China), CEBPB (23431–1-AP, Proteintech, China), ACC1 (21923–1-AP, Proteintech, China), CD36 (18836–1-AP, Proteintech, China), SCD1 (28678–1-AP, Proteintech, China), phosphorylated PI3K (p-PI3K, 138364, Abcam, USA), PI3K (4257, CST, USA), phosphorylated AKT (p-AKT, 66444–1-Ig, Proteintech, China), AKT (10176–2-AP, Proteintech, China), and β-actin (66009–1-Ig, Proteintech, China). Protein bands were visualized using the Tanon 5200 Chemiluminescent Imaging System and quantified using ImageJ software.

### Meta-analysis

The meta-analysis was conducted according to the PRISMA guidelines [79]. PubMed, Embase, Web of Science, and the Cochrane Library were searched from inception to September 2025 for randomized controlled trials comparing Cur with placebo in patients with MASLD and reporting ALT or AST outcomes. Seven eligible studies were included [14–20]. Risk of bias was assessed using the Cochrane risk-of-bias tool (RoB 2). Random-effects models were used to calculate mean differences with 95% confidence intervals, and heterogeneity was assessed using I^2^ and τ^2^. Leave-one-out sensitivity analysis was performed using R (v4.5.1). Detailed Cur administration regimens are provided in Table S18.

### Isolation and identification of *C. sensu*

Fecal samples from HFD-fed rats were suspended in sterile PBS, heated at 80 °C for 20 min to eliminate non-spore-forming bacteria, and enriched in pre-reduced reinforced clostridial medium (RCM) at 37 °C for 36 h. Serially diluted cultures were plated on pre-reduced RCM agar containing 5% defibrinated sheep blood and incubated anaerobically at 37 °C for 48 h. Colonies were purified by repeated anaerobic culture and stored in 20% glycerol at −80 °C. Bacterial identity was determined by 16S rRNA gene sequencing using universal primers 27 F and 1492R, followed by BLASTn comparison and phylogenetic analysis using MEGA 11. The resulting 1,421-bp sequence showed 99.1% identity to *C. sensu* (GenBank accession: NR_104822.1).

### Statistical analysis

Data are presented as the mean ± standard deviation (SD). Statistical analyses were performed using GraphPad Prism 9.5. Differences were assessed using unpaired two-sided Student’s *t*-test, one-way or two-way ANOVA, Kruskal–Wallis test, or permutational multivariate analysis of variance (PERMANOVA), as appropriate. *P* < 0.05 was considered statistically significant.

## Supplementary Information

Supplementary Material 1.

## Data Availability

All relevant data are within the paper and its additional files. The sequencing data generated in this study have been deposited in the Genome Sequence Archive (GSA) at the National Genomics Data Center (NGDC), China National Center for Bioinformation (CNCB). The H3K27ac ChIP-seq, 16S rRNA sequencing, and bulk RNA-seq datasets are available under accession numbers CRA019127, CRA022862, and CRA019123, respectively. Other data and materials are available upon reasonable request.
