# Supplementary material for: Curcumin alleviates metabolic dysfunction-associated steatotic liver disease via gut microbiota-dependent epigenetic alterations
Source: Mol Biomed. 2026 Oct 16;7:214. doi: 10.1186/s43556-026-00606-1 (PMC13675466; doi:10.1186/s43556-026-00606-1)
Supplement: Supplementary file 1 [file 43556_2026_606_MOESM1_ESM.docx]

**Supplementary Information**

# ****Curcumin alleviates metabolic dysfunction-associated steatotic liver disease via gut microbiota-dependent epigenetic alterations****

Yong Zhu^1†^, Jinhu Ma^1,2†^, Danhong Wang^1†^, Mingzhu Cui^1^, Jin Peng^1^, Xuejin Lu^1^, Xingyue Pei^1^, Yi Wang^1^, Jing Lv^1^, Yuxuan Yan^1^, Wenhao Ma^1^, Mingtao Zhao^1^, Shulei You^1^, Jie Ma^1^, Yunshu Tang^3*^& Yaling Zhu^1,3*^

**Authors’ Affiliations:**

^1^Department of Pathophysiology, College of Basic Medical Science, Anhui Medical University, Hefei, Anhui, China.

^2^Department of Hepatobiliary Surgery, State Key Laboratory of Immune Response and Immunotherapy, Centre for Leading Medicine and Advanced Technologies of IHM, The First Affiliated Hospital of USTC, Division of Life Sciences and Medicine, University of Science and Technology of China, Hefei, Anhui, China.

^3^Laboratory Animal Research Center, College of Basic Medical Science, Anhui Medical University, Hefei, Anhui, China.

* **Corresponding author**: Yaling Zhu and Yunshu Tang

E−mail: [zhuyaling@ahmu.edu.cn](mailto:zhuyaling@ahmu.edu.cn;) (Yaling Zhu), [tangyunshu@ahmu.edu.cn](mailto:tangyunshu@ahmu.edu.cn) (Yunshu Tang)

**This Docx file includes:**

Supplementary Figure S1 to S13

Supplementary Table S1 to S18

**Supplementary Figures**

**Supplementary Figure S1. Methodological and robustness evaluations of the meta-analysis.** (a) PRISMA flow diagram illustrating the process of study searching and selection (n = 7 studies). (b) Authors’ judgments about each risk of bias item in every included study (n = 7 studies). (c) Authors’ judgments about each risk of bias item presented as percentages across all included studies (n = 7 studies). (d) Sensitivity analysis indicating no single study unduly influenced the overall ALT effect (n = 7 studies). (e) Sensitivity analysis indicating no single study unduly influenced the overall AST effect (n = 7 studies).

**Supplementary Figure S2. Cur administration showed low oral bioavailability and was metabolized and cleared quickly.** The concentration - time curves of Cur in plasma and colonic tissues were determined after oral administration (80 mg/kg/day) to rats in the Cur group at the initiation of the 4th week. Each point represents the mean ± standard deviation (n = 3).

**Supplementary Figure S3. Fecal microbiota transplantation from Cur-treated rats alleviates HFD-induced mitochondrial structural damage of liver mitochondria in rat.** (a) Representative transmission electron microscopy (TEM) micrographs of hepatic mitochondria in rats from the HFD_FMT and HFD+Cur_FMT groups (n = 6). Low-magnification images (5300× magnification, scale bar, 2 μm) display the overall intracellular distribution and morphology of mitochondria; high-magnification images (17500× magnification, scale bar, 500 nm) are used for detailed ultrastructural observation (Red arrowheads mark mitochondrial membrane rupture, green arrow heads mark mitochondrial fragmented and disorganized cristae). (b) Quantitative analysis of mitochondrial cross-sectional area in the HFD_FMT and HFD+Cur_FMT groups (n = 100, unpaired two-tailed Student’s *t*-test). (c) Analysis of mitochondria disorder by comparison the ratio of entire cristae length to the area of the mitochondrial cross section (L_CRIS_/A_MITO_, n = 100, unpaired two-tailed Student’s t-test). All data were represented as the mean ± SD. ****P<*0.001.

**Supplementary Figure S4. Effects of fecal microbiota transplantation from Cur-treated rats on intestinal barrier function in recipient rats.** (a) Representative hematoxylin and eosin-stained sections of colon tissue. (b) Gut permeability measured by appearance of FITC-labeled dextran in plasma (n = 6). (c) Serum LPS concentrations in rats were measured using specific ELISA kits (n = 6). (d) Representative micrographs of immunohistochemical staining for ZO-1, Occludin and Claudin-1 in colon tissues (n = 6). All data were represented as the mean ± SD. **P<*0.05, ***P<*0.01, ****P<*0.001.

### Supplementary Figure S5. Gut microbiota profiling of ND, HFD and Cur rats. (a) Alpha diversity of microbial communities in ND, HFD, and Cur groups, including goods_coverage index, Pielou evenness, ACE index, and Invsimpson index (n = 4). (b) Coordination plot of non-metric multidimensional scaling (NMDS) results of microbial communities among ND, HFD, and Cur groups based on Bray–Curtis dissimilarity (n = 4). K-W (Kruskal-Wallis) test and PERMANOVA were performed for data analysis.

**Supplementary Figure S6. Cur administration modulated gut-liver axis by suppressing *C. sensu*-derived KYN.** (a) Volcano plot of remarkable differentially abundant metabolites between Cur and HFD groups (n = 4). (b) Heatmap of 102 differentially abundant metabolites in liver samples from the HFD and Cur groups (n = 4). (c) Dot plot showing top 10 enriched KEGG terms of differentially expressed metabolites. (d) Heatmap of significantly altered metabolites and relevant pathways. (e) Top 14 predicted metabolites associated with *C. sensu*. (f) Venn diagram illustrates the overlap of differentially abundant metabolites and gut MGene-predicted metabolites, revealing that KYN was the only overlapping metabolite. (g) The relative abundances of KYN in the HFD and Cur groups (n = 4, unpaired two-tailed Student’s t-test). (h-m) Spearman correlation analysis showing positive correlations between hepatic KYN levels and ALT (h), AST (i), TC (j), TG (k), LDL-C (l), and negative correlation with HDL-C (m) (n = 4). The red lines represent linear regression fits, and the shaded areas indicate 95% confidence intervals. Correlation coefficients R and *p-*values are displayed in the upper left or right corner of each panel. (n) Heatmap of Spearman correlation analysis among *C. sensu*, KYN, and clinical metabolic parameters (n = 4). (o) LC-MS analysis of KYN contents derived from *C. sensu* supernatant with indicated times (n = 5), *P* values were calculated with one-way ANOVA followed by Tukey’s test. The abbreviation *C. sensu* represents *Clostridium_sensu_stricto_1*. All data were represented as the mean ± SD. **P<*0.05, ***P<*0.01, ****P<*0.001.

### Supplementary Figure S7. Functional enrichment analysis of differential H3K27ac peaks after Cur administration. (a-b) GO terms enriched by upregulated H3K27ac peaks (a) and downregulated H3K27ac peaks (b) between livers from HFD and ND groups. (c-h) GSEA showing the activated pathways involved in lipid metabolic processes, including “Peroxisome”, “Cytochrome P450”, “AMPK signaling pathway” and “PPAR signaling pathway”, according to H3K27ac ChIP-Seq data using the livers from Cur and HFD rats. (i-k) Motifs enriched in Cur up-regulated enhancers, Cur down-regulated enhancers and Cur specific motifs.

### Supplementary Figure S8. Cur treatment reversed HFD-induced transcriptomic alteration. (a) Sample distribution profiles of RNA-Seq data analyzed by hierarchical clustering (n = 4). (b-d) Volcano of DEGs between HFD and ND groups (b), DEGs between Cur and HFD groups (c) versus DEGs between Cur and ND groups (d), respectively (red, upregulated; green, downregulated) (n = 4). (e) Heatmap of representative genes in livers from ND, HFD and Cur groups (n = 4). (f) Dot plot of GO terms enriched by DEGs between HFD and ND groups. (g) Dot plot of GO terms enriched by DEGs between Cur and HFD groups. (h) Venn diagram indicating the “key genes” (upper panel, 33 overlapped genes that upregulated in HFD livers compared to ND livers and downregulated in Cur livers compared to HFD livers; lower panel, 112 overlapped genes that downregulated in HFD livers compared to ND livers and upregulated in Cur livers compared to HFD livers). (i) Chord showing the biological processes overrepresented by the “key genes” shown in (h).

**Supplementary Figure S9. Results of enrichment analysis based on H3K27ac ChIP-seq data.** (a-b) H3K27ac signal density at the *Cebpb* enhancer region (*Chr3:156,625,975-156,626,736*) (a) and *Cebpb* gene locus (*Chr3:156,397,052-156,398,087*) (b) was increased in the HFD group compared with the ND group, which was markedly attenuated following Cur treatment. (c) Quantification of H3K27ac signal density within the *Cebpb* enhancer region in each group (n = 3, one-way ANOVA + Tukey test). (d) Quantification of H3K27ac modification density at the *Cebpb* gene in each group (n = 3, one-way ANOVA + Tukey test). (e) ChIP-qPCR analysis of H3K27ac fold enrichment relative to input at the *Cebpb* enhancer fragment and negative control genomic region (n = 3, separate one-way ANOVA for each locus). (f-h) Three independent clinical datasets (GSE126484 (f) Normal: n = 14, MASLD: n = 5; GSE124868 (g) Normal: n = 14, MASLD: n = 7; GSE164084 (h) Normal: n = 3, MASH: n = 3) of RNA-Seq showed significantly elevated *Cebpb* expression in MASLD/MASH patients compared to healthy controls. (i) UMAP visualization of GSE174748 single-cell RNA-sequencing data showing the distribution of different cell clusters. (j) Dot plot showing the expression of canonical cell-type marker genes across clusters for cell-type annotation. (k) UMAP visualization of major cell types annotated based on marker gene expression. (l) Average *CEBPB* expression across annotated cell types, showing the highest expression in hepatocytes. (m) Differential significance analysis of *CEBPB* expression between MASLD and control groups across cell types; color indicates -log_10_(FDR), and asterisks indicate statistical significance. (n) UMAP visualization showing the distribution of *CEBPB*-positive cells in control and MASLD groups. (o) Comparison of the proportion of *CEBPB*-positive hepatocytes between control and MASLD groups.

### Supplementary Figure S10. The key transcription factor AhR mediates the expression of the *Cebpb* gene*.* (a) A schematic overview of the process for identifying the core transcription factor of *CEBPB.* (b-c) Sequence conservation analysis of *CEBPB* (b) and *AHR* (c) among human, rat, and mouse based on the UniProt database (<https://www.uniprot.org/>). (d) *AHR* was a key transcription factor of *CEBPB* gene in the liver according to the Cistrome database (accession number: GSM1024799). All Cistrome data have been carefully curated and processed with a streamlined analysis pipeline and evaluated with comprehensive quality control metrics. (e) UMAP visualization showing *AHR* expression in the GSE174748 single-cell RNA-sequencing dataset. (f) Average *AHR* expression across annotated cell types, showing enriched *AHR* expression in hepatocytes. (g) Joint density visualization of *AHR* and *CEBPB* expression in UMAP space, indicating that their co-expression signal is mainly localized within the hepatocyte compartment. (h) Comparison of *AHR* and *CEBPB* expression between control and MASLD groups in hepatocyte *AHR*⁺*CEBPB*⁺ cells. (i) Single-cell correlation analysis of *AHR* and *CEBPB* expression in hepatocyte *AHR*⁺CEBPB⁺ cells from control and MASLD groups.

**Supplementary Figure S11. KYN regulated lipid steatosis in hepatocytes in a *CEBPB*-dependent manner.** (a-d) Representative Bodipy 493/503 fluorescence staining of lipid droplets accumulation and quantitative analysis in HepG2 and Huh-7 cells treated with KYN (30μM) or DMSO and transfected with siRNAs against *CEBPB* (si-*CEBPB*) or negative control siRNA (si-NC) with or without OA (0.6 mM) treatment (Scale bar, 100 μm) (n = 3). (e-f) Quantification of intracellular TG content in HepG2 and Huh-7 cells treated with KYN (30μM) or DMSO transfected with si-*CEBPB* or si-NC with or without OA treatment (0.6 mM) (n = 3). (g-j) Representative ORO staining of lipid droplets accumulation and quantitative analysis in HepG2 and Huh-7 cells treated with KYN (30 µM) or DMSO and transfected with si-*CEBPB* or si-NC, with or without OA (0.6 mM) treatment (Scale bar, 100 µm) (n = 3). (k) The protein levels of lipid transport and synthesis genes (*Acc1, Cd36* and *Scd1*) in KYN (30μM)- or DMSO-treated HepG2 and Huh-7 cells transfected with si-*CEBPB* or si-NC with or without OA treatment (0.6 mM) were assessed by Western blotting (n = 3). Results were shown as mean ± SD. *P* values were calculated with one-way ANOVA followed by Tukey’s test. *: *P*< 0.05; **: *P* < 0.01; ***: *P* < 0.001.

**Supplementary Figure S12. CEBPB Activates PI3K-Akt Pathway to Promote lipid metabolic processes.** (a) The Principal Component Analysis (PCA) plot of si-*CEBPB* (*CEBPB*-depleted Huh-7 cells, n = 3) and si-NC (negative control counterparts, n = 3) for RNA-Seq analysis. (b) Volcano plot of remarkable differentially expressed genes between si-*CEBPB* and si-NC groups (n = 3). (c) Transcription profiles of differentially expressed genes between si-*CEBPB* and si-NC groups (n = 3). (d) The barplot of enriched biological processes of DEGs (differentially expressed genes). The size and color of node represented gene number and p value respectively. (e) Dot plot of enriched KEGG pathways of differential expressing genes. (f) Sankey plot showing top pathways enriched by significantly down-regulated genes in si-*CEBPB* group. Right column shows the *P* value of enriched genes and pathways, where color represents the log_2_Foldchange value, and left column indicates the genes enriched in the pathway. (g) Protein levels of *CEBPB*, p-PI3K, t-PI3K, p-AKT and t-AKT in *CEBPB*-depleting HepG2 and Huh-7 cells with OA treatment (0.6 mM) in the co-culture system were examined by Western blotting (n = 3).

**Supplementary Figure S13. KYN reverses the protective effect of Cur on HFD-induced hepatic CEBPB and AHR expression.** Representative immunofluorescence staining of CEBPB (red), AHR (green), and nuclei (DAPI, blue) in liver sections from rats in the ND, HFD, HFD+Cur, and HFD+Cur+KYN groups. Quantitative analysis of the mean fluorescence intensity (MFI) of CEBPB and AHR is shown on the right (n = 6). All data were represented as the mean ± SD. **P<*0.05, ***P<*0.01, ****P<*0.001.

**Supplementary Tables**

## Supplementary Table S1. The meta-analysis of AST (Aspartate Aminotransferase).

| **Study** | **Curcumin Treated Group** | | | **Control Treated Group** | | | **Weight(%)** | **Mean Difference** |
| --- | --- | --- | --- | --- | --- | --- | --- | --- |
|  | **Mean** | **SD** | **Total** | **Mean** | **SD** | **Total** |  | **MD(95%Cl)** |
| He 2024 | -0.82 | 2.7926 | 40 | 5 | 7.4074 | 40 | 20.70% | -5.82(-8.27, -3.37) |
| Mirhafez 2021 | -5.8 | 4.068 | 22 | -1.4 | 5.38 | 22 | 19.00% | -4.40(-7.22, -1.58) |
| Mirhafez 2021(2) | -5.5 | 14.5 | 35 | 2.5 | 10.5 | 37 | 8.80% | -8.00(-13.88, -2.12) |
| Hariri 2020 | -2.03 | 11.9896 | 23 | 1.1 | 8.4236 | 22 | 8.50% | -3.13(-9.16, 2.90) |
| Mirhafez 2019 | -2 | 10.6148 | 32 | 2.44 | 8.65926 | 27 | 11.10% | -4.44(-9.36, 0.48) |
| Panahi 2017 | -6.95 | 7.47 | 44 | 3.79 | 6.43 | 43 | 18.50% | -10.74(-13.67, -7.81) |
| Rahmani 2016 | -5.04 | 6.49 | 40 | 2.02 | 11.79 | 40 | 13.40% | -7.06(-11.23, -2.89) |
| **Total(95% Cl)** |  |  | **236** |  |  | **231** | **1** | **-6.44(-8.52, -4.35)** |

## Supplementary Table S2. The meta-analysis of ALT (Alanine transaminase).

| **Study** | **Curcumin Treated Group** | | | **Control Treated Group** | | | **Weight(%)** | **Mean Difference** |
| --- | --- | --- | --- | --- | --- | --- | --- | --- |
|  | **Mean** | **SD** | **Total** | **Mean** | **SD** | **Total** |  | **MD(95%Cl)** |
| He 2024 | -0.88 | 3.889 | 40 | 7.1 | 11.51 | 40 | 0.279% | -7.98（-11.75, -4.21) |
| Mirhafez 2021 | -0.82 | 4.51 | 22 | 5 | 20.96 | 22 | 0.125% | -5.82(-14.78, 3.14) |
| Mirhafez 2021(2) | -5.7 | 25 | 35 | -1.3 | 25.5 | 37 | 0.085% | -4.40(-16.07, 7.27) |
| Hariri 2020 | -2.82 | 22.5477 | 23 | -0.46 | 16.4882 | 22 | 0.087% | -2.36(-13.87, 9.15) |
| Mirhafez 2019 | -6.12 | 19.7037 | 32 | 1.22 | 11.822 | 27 | 0.141% | -7.34(-15.49, 0.81) |
| Panahi 2017 | -10.61 | 15.49 | 44 | 4.51 | 7.4 | 43 | 0.229% | -15.12(-20.20, -10.04) |
| Rahmani 2016 | -2.99 | 47.38 | 40 | -1.62 | 12.3 | 40 | 0.055% | -1.37(-16.54, 13.80) |
| **Total(95% Cl)** |  |  | **236** |  |  | **231** | **100%** | **-8.10(-11.95, -4.24)** |

## Supplementary Table S3. The top enriched taxas in ND, HFD and Cur groups, respectively.

| **Taxa** | **Group** | **LDA** | ***P*-value** |
| --- | --- | --- | --- |
| k__Bacteria\|p__Bacteroidota | ND | 4.95 | 2.45E-02 |
| k__Bacteria\|p__Bacteroidota\|c__Bacteroidia | ND | 4.95 | 2.45E-02 |
| k__Bacteria\|p__Bacteroidota\|c__Bacteroidia\|o__Bacteroidales | ND | 4.95 | 2.31E-02 |
| k__Bacteria\|p__Firmicutes\|c__Clostridia\|o__Lachnospirales\|f__Lachnospiraceae\|g__Lachnospiraceae_NK4A136_group | ND | 4.66 | 1.83E-02 |
| k__Bacteria\|p__Firmicutes\|c__Clostridia\|o__Lachnospirales\|f__Lachnospiraceae\|g__Lachnospiraceae_NK4A136_group\|s__Trichinella_pseudospiralis | ND | 4.48 | 1.54E-02 |
| k__Bacteria\|p__Firmicutes\|c__Bacilli\|o__Lactobacillales\|f__Lactobacillaceae\|g__Lactobacillus\|s__Lactobacillus_johnsonii | ND | 4.23 | 1.83E-02 |
| k__Bacteria\|p__Firmicutes\|c__Bacilli\|o__Lactobacillales\|f__Lactobacillaceae\|g__Lactobacillus | ND | 4.22 | 4.88E-02 |
| k__Bacteria\|p__Bacteroidota\|c__Bacteroidia\|o__Bacteroidales\|f__Tannerellaceae | ND | 4.16 | 3.73E-02 |
| k__Bacteria\|p__Bacteroidota\|c__Bacteroidia\|o__Bacteroidales\|f__Tannerellaceae\|g__Parabacteroides | ND | 4.16 | 3.88E-02 |
| k__Bacteria\|p__Firmicutes\|c__Clostridia\|o__Lachnospirales\|f__Lachnospiraceae\|g__[Eubacterium]_xylanophilum_group | ND | 3.67 | 3.45E-02 |
| k__Bacteria\|p__Bacteroidota | HFD | 5.259399 | 2.10E-02 |
| k__Bacteria\|p__Firmicutes | HFD | 5.029224 | 2.96E-02 |
| k__Bacteria\|p__Firmicutes\|c__Clostridia\|o__Peptostreptococcales-Tissierellales | HFD | 5.028144 | 2.96E-02 |
| k__Bacteria\|p__Firmicutes\|c__Clostridia\|o__Peptostreptococcales-Tissierellales\|f__Peptostreptococcaceae | HFD | 5.027476 | 2.96E-02 |
| k__Bacteria\|p__Firmicutes\|c__Clostridia\|o__Peptostreptococcales-Tissierellales\|f__Peptostreptococcaceae\|g__Romboutsia | HFD | 5.027432 | 2.96E-02 |
| k__Bacteria\|p__Firmicutes\|c__Clostridia\|o__Peptostreptococcales-Tissierellales\|f__Peptostreptococcaceae\|g__Romboutsia\|s__Romboutsia_ilealis | HFD | 4.806679 | 3.73E-02 |
| k__Bacteria\|p__Firmicutes\|c__Bacilli | HFD | 4.74207 | 7.28E-03 |
| k__Bacteria\|p__Firmicutes\|c__Clostridia\|o__Clostridiales | HFD | 4.74207 | 7.28E-03 |
| k__Bacteria\|p__Firmicutes\|c__Clostridia\|o__Clostridiales\|f__Clostridiaceae | HFD | 4.741806 | 7.28E-03 |
| k__Bacteria\|p__Firmicutes\|c__Clostridia\|o__Clostridiales\|f__Clostridiaceae\|g__Clostridium_sensu_stricto_1 | HFD | 4.635212 | 3.45E-02 |
| k__Bacteria\|p__Firmicutes\|c__Bacilli\|o__Staphylococcales | Cur | 5.29 | 2.64E-02 |
| k__Bacteria\|p__Verrucomicrobiota\|c__Verrucomicrobiae\|o__Verrucomicrobiales\|f__Akkermansiaceae | Cur | 5.29 | 2.64E-02 |
| k__Bacteria\|p__Verrucomicrobiota\|c__Verrucomicrobiae\|o__Verrucomicrobiales\|f__Akkermansiaceae\|g__Akkermansia | Cur | 5.29 | 2.64E-02 |
| k__Bacteria\|p__Verrucomicrobiota\|c__Verrucomicrobiae\|o__Verrucomicrobiales\|f__Akkermansiaceae\|g__Akkermansia\|s__Akkermansia_muciniphila | Cur | 5.29 | 2.64E-02 |
| k__Bacteria\|p__Verrucomicrobiota\|c__Verrucomicrobiae | Cur | 5.29 | 2.64E-02 |
| k__Bacteria\|p__Verrucomicrobiota | Cur | 5.29 | 2.64E-02 |
| k__Bacteria\|p__Firmicutes\|c__Clostridia\|o__Oscillospirales | Cur | 4.83 | 1.73E-02 |
| k__Bacteria\|p__Firmicutes\|c__Clostridia\|o__Oscillospirales\|f__[Eubacterium]_coprostanoligenes_group | Cur | 4.81 | 2.10E-02 |
| k__Bacteria\|p__Firmicutes\|c__Bacilli\|o__Erysipelotrichales\|f__Erysipelotrichaceae\|g__Faecalitalea | Cur | 4.28 | 2.49E-02 |
| k__Bacteria\|p__Firmicutes\|c__Clostridia\|o__Lachnospirales\|f__Lachnospiraceae\|g__Blautia | Cur | 4.03 | 2.10E-02 |

## Supplementary Table S4. The differential metabolites between Cur and HFD livers.

| **epsilon-(gamma-L-Glutamyl)-L-lysine** | **Type** | **Log2fc** | ***P*-value** | **VIP** |
| --- | --- | --- | --- | --- |
| Cyclobutyl fentanyl | up | 0.34 | 3.42E-02 | 2.21 |
| MLS000862193-01 | up | 0.27 | 5.27E-02 | 2.12 |
| MLS001148235-01 | up | 0.26 | 8.50E-03 | 2.25 |
| cis-4,5-Dihydroxy-4,5-dihydropyrene | up | 0.23 | 6.06E-02 | 2.18 |
| MLS001140990-01 | up | 0.21 | 8.45E-02 | 2.01 |
| Imazapic | up | 0.20 | 6.53E-02 | 2.08 |
| trans-Aconitic acid | up | 0.20 | 4.87E-03 | 2.41 |
| TGX-221 | up | 0.19 | 4.59E-02 | 2.22 |
| 2,4-dimethyl-5-oxohexanoic acid | up | 0.17 | 1.48E-02 | 2.50 |
| D-xylopyranoside | up | 0.17 | 1.69E-02 | 2.23 |
| Prostaglandin H2 | up | 0.15 | 5.87E-02 | 2.08 |
| Chlorogenic acid | up | 0.14 | 4.24E-02 | 2.21 |
| (S)-3-Hydroxy-N-methylcoclaurine | up | 0.14 | 1.02E-01 | 2.01 |
| NP-015145 | up | 0.14 | 2.73E-02 | 2.35 |
| QUINOXALINECARBOXYLIC ACID | up | 0.13 | 4.95E-02 | 2.05 |
| Hecogenin | up | 0.13 | 3.33E-02 | 2.05 |
| Anhydrobrazilic Acid | up | 0.12 | 8.20E-03 | 2.30 |
| 11-O-Demethyl-17-O-deacetylvindoline | up | 0.12 | 2.91E-02 | 2.15 |
| Alpha-Linolenoyl ethanolamide | up | 0.12 | 6.51E-02 | 2.08 |
| Atraton | up | 0.12 | 5.55E-02 | 2.06 |
| GW 0742 | up | 0.11 | 6.02E-02 | 2.02 |
| N-AMIDINO-L-ASPARTATE | up | 0.11 | 4.60E-02 | 2.13 |
| (R)-Higenamine | up | 0.10 | 4.95E-02 | 2.02 |
| Sodium gluconate | up | 0.10 | 6.21E-02 | 2.12 |
| 4-Hydroxycinnamic acid | up | 0.10 | 2.48E-03 | 2.49 |
| Ingenol-3-angelate | up | 0.10 | 2.87E-02 | 2.22 |
| Fenoprofen | up | 0.10 | 5.10E-02 | 2.06 |
| O-Demethylpuromycin | up | 0.10 | 6.46E-02 | 2.04 |
| cyclopenta[a]phenanthren-17-ol | up | 0.09 | 1.07E-02 | 2.37 |
| Fluazifop | up | 0.09 | 1.66E-02 | 2.28 |
| 3-(3,4-Dihydroxyphenyl)pyruvate | up | 0.09 | 7.12E-02 | 2.06 |
| 20a,22b-Dihydroxycholesterol | up | 0.08 | 5.73E-02 | 2.20 |
| Bilobalide A | up | 0.08 | 1.69E-02 | 2.26 |
| Harmaline | up | 0.08 | 1.47E-02 | 2.22 |
| epsilon-(gamma-L-Glutamyl)-L-lysine | up | 0.08 | 9.10E-03 | 2.56 |
| 5,7-dimethoxy-2H-chromen-2-one | up | 0.08 | 5.57E-02 | 2.01 |
| NP-004713 | up | 0.06 | 2.93E-02 | 2.02 |
| Nudicauline | up | 0.06 | 4.07E-02 | 2.09 |
| 1-Dodecyl-2-pyrrolidinone | up | 0.05 | 7.98E-02 | 2.06 |
| Leucocrystal violet | up | 0.05 | 1.70E-02 | 2.17 |
| NCGC00385245-01 | up | 0.05 | 1.49E-02 | 2.28 |
| Dimethylglycine | up | 0.05 | 7.04E-02 | 2.01 |
| 2-[5-(2-hydroxypropyl)oxolan-2-yl]propanoic acid | up | 0.05 | 8.45E-03 | 2.25 |
| Maltotetraose | up | 0.05 | 5.53E-02 | 2.01 |
| Glycineamideribotide | up | 0.04 | 4.40E-02 | 2.10 |
| NP-020439 | up | 0.04 | 8.88E-02 | 2.04 |
| 2,2'-(3-methylcyclohexane-1,1-diyl)diacetic acid | up | 0.04 | 5.31E-02 | 2.08 |
| 2-Carboxy-D-arabinitol 1-phosphate | up | 0.04 | 1.12E-02 | 2.28 |
| S-Nitroso-L-glutathione | up | 0.04 | 5.00E-02 | 2.04 |
| NP-021733 | up | 0.02 | 2.17E-02 | 2.15 |
| carboxylic acid | down | -0.02 | 5.58E-02 | 2.01 |
| Kynurenine | down | -0.02 | 6.16E-02 | 2.03 |
| DL-2-Aminoadipic acid | down | -0.02 | 6.31E-02 | 2.02 |
| 5-Fluoro THJ | down | -0.03 | 4.81E-02 | 2.15 |
| MMV687765 | down | -0.04 | 6.58E-02 | 2.16 |
| L-Lysine | down | -0.04 | 4.01E-02 | 2.09 |
| N-(5-acetamidopentyl)acetamide | down | -0.04 | 1.20E-02 | 2.16 |
| Allocystathionine | down | -0.04 | 6.16E-03 | 2.43 |
| 3-Dehydroshikimate | down | -0.05 | 6.41E-02 | 2.08 |
| 4a-Carbinolamine tetrahydrobiopterin | down | -0.05 | 6.99E-03 | 2.50 |
| Caffeine | down | -0.05 | 6.63E-02 | 2.12 |
| Mepivacaine | down | -0.05 | 1.62E-02 | 2.20 |
| LY 294002 | down | -0.05 | 5.73E-02 | 2.06 |
| Sepiapterin | down | -0.06 | 4.99E-02 | 2.15 |
| AMP | down | -0.06 | 2.07E-02 | 2.31 |
| 1-Deoxynojirimycin | down | -0.07 | 7.38E-02 | 2.07 |
| (R,S)-Norlaudanosoline | down | -0.07 | 7.45E-02 | 2.02 |
| Triphenyl phosphate | down | -0.07 | 5.17E-03 | 2.53 |
| Hydroxyatrazine | down | -0.07 | 5.29E-02 | 2.08 |
| Oxymorphone | down | -0.07 | 4.98E-02 | 2.17 |
| Arachidonoyl thio-PC | down | -0.07 | 2.77E-02 | 2.19 |
| 9-cis-Retinol | down | -0.08 | 5.60E-02 | 2.11 |
| 2-Amino-4-chloro-6-methylpyrimidine | down | -0.08 | 3.24E-02 | 2.11 |
| MEVALOLACTONE | down | -0.09 | 2.98E-02 | 2.05 |
| NCGC00160318-01 | down | -0.10 | 1.70E-02 | 2.39 |
| MLS001148102-01 | down | -0.10 | 9.19E-02 | 2.08 |
| O-trimethoxy-3,4,5-benzoyl-OH-vincamajine | down | -0.11 | 6.11E-02 | 2.04 |
| 2-Acetamidofluorene | down | -0.12 | 1.76E-02 | 2.17 |
| Porritoxinol | down | -0.12 | 5.00E-02 | 2.06 |
| NBD-Stearoyl-2-Arachidonoyl-sn-glycerol | down | -0.12 | 6.34E-04 | 2.60 |
| Midazolam | down | -0.13 | 4.12E-02 | 2.13 |
| Protoporphyrinogen IX | down | -0.14 | 1.64E-02 | 2.23 |
| Idebenone | down | -0.14 | 1.54E-02 | 2.10 |
| Dihydrojasmone | down | -0.14 | 5.41E-02 | 2.01 |
| 3alpha,12alpha-Dihydroxy-5beta-chol-6-enoate | down | -0.15 | 4.46E-02 | 2.04 |
| MG(18_2(9Z,12Z)_0_0_0_0)[rac] | down | -0.15 | 4.21E-02 | 2.24 |
| Delphinidin 3-O-3'',6''-O-dimalonylglucoside | down | -0.16 | 2.90E-03 | 2.50 |
| Strophanthidin | down | -0.16 | 2.14E-02 | 2.28 |
| andrimid | down | -0.18 | 5.79E-04 | 2.62 |
| Beauvericin | down | -0.18 | 2.65E-02 | 2.33 |
| MMV000907 | down | -0.19 | 6.98E-02 | 2.02 |
| MLS001140512-01 | down | -0.19 | 4.77E-02 | 2.14 |
| F-Dehydroxy-Albicidin | down | -0.20 | 5.85E-03 | 2.36 |
| 2,6-Dimethoxy-4-pyrimidinyl | down | -0.20 | 2.91E-02 | 2.17 |
| O-1821 | down | -0.20 | 4.56E-02 | 2.08 |
| Prostaglandin E2-biotin | down | -0.21 | 9.34E-03 | 2.58 |
| PIPEMIDIC ACID | down | -0.22 | 1.62E-04 | 2.66 |
| Scytophycin C | down | -0.22 | 1.22E-02 | 2.35 |
| Salicylamide | down | -0.23 | 7.69E-02 | 2.02 |
| Tamsulosin | down | -0.23 | 4.59E-02 | 2.11 |
| Flavin adenine dinucleotide disodium | down | -0.31 | 6.55E-02 | 2.07 |
| Lathosterol | down | -0.34 | 2.84E-03 | 2.62 |

## Supplementary Table S5. The metabolites of *Clostridium*.

| Index | PMID | Metabolite | Metabolite PubChem CID | Metabolite ChEBI | Metabolite Metabolights | Associative mode | human/mouse | Sample | Measurement technique |
| --- | --- | --- | --- | --- | --- | --- | --- | --- | --- |
| 4 | 31780788 | 3-Phenylpropionic acid | 107 | CHEBI:28631 | MTBLC28631 | correlatively | mouse | cecal contents | LC/MS analysis |
| 4 | 31780788 | Benzoic Acid | 243 | CHEBI:30746 | MTBLC30746 | correlatively | mouse | cecal contents | LC/MS analysis |
| 11 | 31214133 | Hypoxanthine | 135398638 | CHEBI:17368 |  | correlatively | mouse | fecal | 16S rRNA gene sequences, UPLC-Q-TOF/MS analysis |
| 39 | 30359203 | Butyrate | 104775 | CHEBI:17968 | MTBLC17968 | causally | human | fecal | 16S rRNA sequence |
| 72 | 22325238 | Pyruvate | 107735 | CHEBI:15361 | MTBLC15361 | causally | human |  |  |
| 72 | 22325238 | Acetyl phosphate(2-) | 4183249 | CHEBI:22191 | MTBLC22191 | causally | human |  |  |
| 204 | 34346464 | Butyrate | 104775 | CHEBI:17968 | MTBLC17968 | causally | human | fecal | GC analysis |
| 213 | 37471289 | Bile acid | 439520 | CHEBI:22868 |  | correlatively | mouse | fecal | LC/MS analysis |
| 240 | 36076430 | Butyrate | 104775 | CHEBI:17968 | MTBLC17968 | correlatively | human | fecal | LC/MS analysis |
| 258 | 36638362 | Deoxycholic Acid | 222528 | CHEBI:28834 | MTBLC28834 | correlatively | mouse | fecal | UHPLC-QQQ-MS analysis |
| 265 | 37784018 | D-(+)-Cellobiose | 10712 | CHEBI:36217 | MTBLC36217 | correlatively | mouse | plasma | LC-MS/MS analysis |
| 301 | 37399653 | Amylotriose | 439586 | CHEBI:140999 |  | correlatively | human | fecal | LC-MS analysis |
| **305** | **37450589** | **Kynurenine** | **846** | **CHEBI:28683** | **MTBLC28683** | **correlatively** | **mouse** | **plasma** | **Metagenomics and untargeted metabolomics joint analyses** |
| 326 | 36076453 | 4-Hydroxybenzoic acid | 135 | CHEBI:30763 | MTBLC30763 | causally | human | fecal | UHPLC/HR-MS analysis |

## Supplementary Table S6. The top 10 hypo- and hyper-acetylated peaks identified by ChIP-Seq in HFD rats (Ranked by Log_2_FoldChange).

| **Chr.** | **Position (Start-End)** | **log_2_FoldChange** | ***P*-value** | **Padj** | **Regulation** |
| --- | --- | --- | --- | --- | --- |
| Hypoacetylated peaks | | | | | |
| 6 | 6:135838144-135838445 | -3.85 | 3.19E-12 | 2.85E-08 | Down |
| 2 | 2:225872747-225873426 | -2.62 | 1.21E-08 | 2.06E-05 | Down |
| 1 | 1:85509682-85510005 | -2.55 | 4.89E-09 | 1.34E-05 | Down |
| 10 | 10:110003943-110005522 | -2.52 | 1.01E-11 | 7.25E-08 | Down |
| 1 | 1:226177771-226180178 | -2.32 | 3.27E-16 | 5.84E-12 | Down |
| 10 | 10:110012167-110014342 | -2.26 | 1.88E-08 | 2.90E-05 | Down |
| 1 | 1:226234272-226236070 | -1.99 | 6.43E-09 | 1.53E-05 | Down |
| 19 | 19:49699281-49701873 | -1.97 | 3.53E-11 | 2.00E-07 | Down |
| 15 | 15:36020345-36021020 | -1.8 | 2.68E-08 | 3.54E-05 | Down |
| 1 | 1:226149479-226153282 | -1.67 | 6.02E-08 | 7.21E-05 | Down |
| Hyperacetylated peaks | | | | | |
| 14 | 14:22683513-22686302 | 8.46 | 5.60E-09 | 1.43E-05 | Up |
| X | X:18161404-18164639 | 4.43 | 2.16E-19 | 7.71E-15 | Up |
| 13 | 13:12174022-12175500 | 3.58 | 5.64E-15 | 6.72E-11 | Up |
| 13 | 13:6663028-6665462 | 3.25 | 3.91E-11 | 2.00E-07 | Up |
| 1 | 1:24903573-24904257 | 2.83 | 3.90E-09 | 1.16E-05 | Up |
| 10 | 10:31626071-31627586 | 2.57 | 8.22E-10 | 2.94E-06 | Up |
| 4 | 4:50938308-50939224 | 2.43 | 1.21E-10 | 5.41E-07 | Up |
| 9 | 9:7875787-7877157 | 2.39 | 2.61E-10 | 1.03E-06 | Up |
| 15 | 15:107951292-107954372 | 2.33 | 1.66E-09 | 5.39E-06 | Up |
| 17 | 17:75238805-75241216 | 2.19 | 9.27E-09 | 1.98E-05 | Up |

## Supplementary Table S7. The top 10 hypo- and hyper-acetylated peaks identified by ChIP-Seq in Cur livers compared to HFD livers (Ranked by Log_2_FoldChange).

| **Chr.** | **Position (Start-End)** | **log_2_FoldChange** | ***P*-value** | **Padj** | **Regulation** |
| --- | --- | --- | --- | --- | --- |
| Hypoacetylated peaks | | | | | |
| 1 | 148642090-148644556 | -5.07 | 1.70E-07 | 1.81E-04 | Down |
| 14 | 448090-448390 | -3.53 | 5.01E-09 | 1.44E-05 | Down |
| 15 | 24396554-24397338 | -2.85 | 3.01E-08 | 6.33E-05 | Down |
| 1 | 11930262-11930720 | -2.79 | 4.28E-08 | 7.61E-05 | Down |
| 1 | 206759008-206762417 | -2.73 | 1.86E-07 | 1.81E-04 | Down |
| 14 | 46580044-46580785 | -2.64 | 3.45E-07 | 2.87E-04 | Down |
| 10 | 31626071-31627586 | -2.55 | 3.37E-09 | 1.34E-05 | Down |
| 20 | 29586114-29587621 | -2.29 | 2.91E-07 | 2.57E-04 | Down |
| 5 | 91125016-91125850 | -2.28 | 5.63E-07 | 4.20E-04 | Down |
| 12 | 1250279-1251023 | -2.25 | 4.39E-08 | 7.61E-05 | Down |
| Hyperacetylated peaks | | | | | |
| 2 | 179949208-179975382 | 1.45 | 2.67E-09 | 1.19E-05 | Up |
| 5 | 128778866-128782695 | 1.72 | 5.24E-09 | 1.44E-05 | Up |
| 17 | 69427468-69436026 | 1.88 | 1.98E-10 | 1.29E-06 | Up |
| 6 | 37476256-37477741 | 2.06 | 4.73E-14 | 8.45E-10 | Up |
| 1 | 167886023-167887546 | 2.25 | 1.60E-14 | 5.70E-10 | Up |
| 18 | 32342850-32353608 | 1.75 | 5.24E-12 | 4.68E-08 | Up |
| 14 | 22717102-22727690 | 2.16 | 4.02E-09 | 1.44E-05 | Up |
| 8 | 38715531-38717703 | 2.22 | 2.16E-10 | 1.29E-06 | Up |
| 4 | 71602538-71606692 | 1.83 | 4.81E-10 | 2.45E-06 | Up |
| 5 | 52673102-52687970 | 1.94 | 4.83E-09 | 1.44E-05 | Up |

## Supplementary Table S8. Motifs enriched in Cur up-regulated enhancers and functions.

| **Best match** | **Function** | **Reference** |
| --- | --- | --- |
| RARa | ①Regulation of apoptosis;②Induction of cholesterol efflux; ③Inhibition of inflammation | PMID:24833708；PMID:37591326；PMID:36291054 |
| HNF1b | ①Hepatocyte nuclear factor 1β; ②Inhibition of fatty liver | PMID:28942246；PMID:37961802；PMID:29879443；PMID:32623698 |
| ATHB-16 | Inhibition of cell expansion during plant development | PMID:11726705 |
| RORgt | A signature transcription factor for Th17 cell formation | PMID:33790913 |
| BH1 | Inhibition of apoptosis | PMID:35358925 |

## Supplementary Table S9. Motifs enriched in Cur down-regulated enhancers and functions.

| **Best match** | **Function** | **Reference** |
| --- | --- | --- |
| LEC2 | Initiated plant embryo development | PMID:35318532 |
| MOT2 | ①Alga Chlamydomonas reinhardtii type 2 Mo transporter protein; ②Enabling ubiquitin-protein ligase activity; ③Involved in cellular macromolecular catabolic processes. | PMID:23132250 |
| An_0287 | / | / |
| NKX3-1 | ①Negative regulators of epithelial cell growth in prostate tissue; ②Potential markers of increased risk of occult lymph node metastasis and poor prognosis in oral squamous cell carcinoma | PMID:31865345；PMID:22344708 |
| MATALPHA2 | ①Enabling DNA binding activity; ②enhanced expression in hepatocellular carcinoma;③ induction of steatosis | PMID:23814050；PMID:25075345；PMID:26858410 |

## Supplementary Table S10. Cur specific motifs and functions

| **Best match** | **Function** | **Reference** |
| --- | --- | --- |
| NRF1 | ①Promoted mitochondrial function and mitochondrial DNA replication; ②Inhibited lipid cytokines; ③Anti-oxidant | PMID:35308224；PMID:31430204；PMID:38173037；PMID:37625288；PMID:18826952；PMID:37625288 |
| MATR3 | Promoted development of HCC, lung cancer, depression | PMID:35255005；PMID:34090445；PMID:34633764 |
| CDF3 | Regulators of the nitrate response | PMID:33329669 |
| FOXH1 | Promoted cell growth and cell migration/invasion and thus HCC development | PMID:35255005 |
| Nr2e3 | ①Promoted transcription complex formation; Reduced expression in cirrhotic and liver tumor tissues; ②Maintains basal endoplasmic reticulum expression | PMID:28878246；PMID:30991008；PMID:38790135；PMID:26149760 |

## Supplementary Table S11. The top 10 up- and down- regulated genes examined by RNA-Seq of HFD and ND livers (Ranked by Log2FoldChange).

| **Genes** | **Log_2_FoldChange** | ***P*-value** | **Function** |
| --- | --- | --- | --- |
| Up -regulation | | | |
| *Tbx3* | 2.3 | 3.18E-08 | Involved in developmental processes, limb pattern formation and branching morphogenesis |
| *Vwf* | 1.89 | 6.43E-08 | Promoted adhesion of platelets to the sites; a chaperone for coagulation factor VIII |
| *Fmo1* | 1.78 | 4.79E-08 | ①Involved in cell volume regulation;② catalyzed the oxygenation of nitrogen- and sulfur-containing compounds |
| *Cd74* | 1.72 | 2.11E-07 | ①Involved in MHC class II antigen processing; ②antiviral activity |
| *Gstm7* | 1.35 | 1.21E-07 | Conjugation of reduced glutathione to a wide number of exogenous and endogenous hydrophobic electrophiles |
| *Rt1-da* | 1.34 | 1.33E-07 | ①MHC class II protein complex binding activity;② T cell receptor binding activity; ③involved in antigen processing |
| *Gstm7* | 1.34 | 9.63E-08 | ①Anion binding activity;② glutathione peroxidase activity;③ involved in cellular detoxification of nitrogen compound |
| *Idh2* | 1.31 | 2.43E-10 | Associated or interacted with the pyruvate dehydrogenase |
| *Tmem37* | 1.29 | 1.47E-07 | Involved in calcium ion transmembrane transport and regulation of ion transmembrane transport |
| *Ptms* | 1.28 | 1.95E-07 | Involved in DNA replication; |
| Down -regulation | | | |
| *Loc100362027* | -3 | 7.76E-11 | Encodes a ribosomal protein; involved in peptide chain elongation and rRNA processing in the nucleus and cytosol |
| *Rps18* | -2.74 | 5.04E-10 | Synthesis of proteins in the cell |
| *Rps20* | -2.7 | 7.78E-10 | Synthesis of proteins in the cell |
| *Rps16* | -2.53 | 1.51E-11 | Synthesis of proteins in the cell |
| *Rfc4* | -2.37 | 1.49E-09 | Involved in the elongation of the multiprimed DNA template |
| *Rpl27a* | -2.32 | 1.72E-09 | Component of the large ribosomal subunit; synthesis of proteins in the cell |
| *Coq10b* | -2.12 | 5.97E-10 | Involved in cellular respiration and ubiquinone biosynthetic process |
| *Eif4a2* | -1.62 | 3.55E-11 | Involved in cap recognition and negative regulation of RNA-directed 5'-3' RNA polymerase activity |
| *Loc100360449* | -2.98 | 1.31E-11 | \ |
| *Loc100365810* | -2.75 | 5.12E-10 | \ |

## Supplementary Table S12. The top 10 up- and down- regulated genes examined by RNA-Seq of Cur and HFD livers (Ranked by Log2FoldChange).

| **Genes** | **Log_2_FoldChange** | **P-value** | **Function** |
| --- | --- | --- | --- |
| Up -regulation | | | |
| *Akr1c2* | 4.58 | 7.00E-06 | Involved in synthesis of bile acids and bile salts and Metabolism |
| *Amhr2* | 2.21 | 2.90E-04 | Resulted in male sex differentiation |
| *Gmnn* | 2.18 | 6.49E-05 | Inhibited DNA replication and transcriptional activity of a subset of Hox proteins |
| *Crym* | 1.56 | 1.71E-05 | ①Catalyzed the reduction of imine bonds in brain substrates; ②bound thyroid hormone |
| *Miox* | 1.45 | 1.16E-06 | ①Enables ferric iron binding activity and inositol oxygenase activity;② involved in inositol catabolic process. |
| *Serpina7* | 1.41 | 1.63E-04 | Encoded the major thyroid hormone transport protein, TBG, in serum |
| *Dapp1* | 1.27 | 2.05E-04 | Involved in signal transduction; regulated B-cell antigen receptor (BCR)-signaling downstream of PI3K |
| *Birc2* | 1.22 | 2.93E-04 | ①Regulated caspases and apoptosis;② modulated inflammatory signaling and immunity, mitogenic kinase signaling |
| *Loc100911417* | 3.01 | 1.76E-05 | \ |
| *Aabr07061378.1* | 2.15 | 5.73E-05 | \ |
| Down -regulation | | | |
| *Dync2li1* | -4.42 | 5.96E-04 | ①Facilitated the assembly of organelles; ②involved in the regulation of ciliary length |
| *Ebf4* | -4.41 | 1.52E-03 | ①Transcription factor; ②regulated FAS/CD95-mediated apoptosis in cytotoxic NK cells and T-cells |
| *Popdc2* | -2.06 | 9.76E-04 | ①Increased cell surface expression of the potassium channel KCNK2; ②enhanced current density |
| *Rgs16* | -1.87 | 3.25E-04 | Regulated G protein-coupled receptor signaling cascades; inhibited signal transduction |
| *Ppard* | -1.61 | 2.51E-04 | ①Improved insulin sensitivity, glucose homeostasis, and lipid metabolism; ②reduced inflammation |
| *Tor3a* | -1.61 | 3.66E-04 | Enabled ATP binding activity |
| *Nr0b2* | -1.6 | 1.46E-03 | A negative regulator of receptor-dependent signaling pathways; inhibited estrogen receptor |
| *Cyp4a8* | -1.46 | 2.85E-04 | ①Involved in fatty acid metabolic process; enables fatty acid omega-hydroxylase activity； ②kidney development |
| *Col4a2* | -1.21 | 1.22E-03 | ①Inhibited proliferation and migration of endothelial cells; ②reduced mitochondrial membrane potential and apoptosis |
| *Sez6* | -1.2 | 4.91E-04 | Involved in cell-cell recognition and in neuronal membrane signaling |

## Supplementary Table S13. The top 10 up- and down- regulated genes examined by RNA-Seq of Cur and ND livers (Ranked by Log_2_FoldChange).

| Genes | Log_2_FoldChange | P-value | Function |
| --- | --- | --- | --- |
| Up -regulation | | | |
| *Ca3* | 1.7 | 6.76E-06 | Reversible hydration of carbon dioxide |
| *Csad* | 1.62 | 3.03E-06 | Catalyzed the decarboxylation of cysteinesulfinate to hypotaurine |
| *Fmo1* | 1.56 | 1.67E-06 | Catalyzed the oxygenation of a wide variety of nitrogen- and sulfur-containing compounds including xenobiotics |
| *Cyp1a2* | 1.41 | 3.30E-05 | ①Involved in the metabolism of various endogenous substrates, including fatty acids, steroid hormones and vitamins;②catalyzed the hydroxylation of carbon-hydrogen bonds |
| *Ces1d* | 1.4 | 3.08E-06 | ①Involved in the detoxification of xenobiotics and in the activation of ester and amide prodrugs; ②participated in fatty acyl and cholesterol ester metabolism |
| *Ephx1* | 1.18 | 2.05E-04 | Involved in the metabolism of endogenous lipids and the metabolism of endogenous lipids |
| *Idh2* | 1.1 | 8.67E-08 | ①Involved in intermediary metabolism and energy production; ②alternative splicing results in multiple transcript variants |
| *Dio1* | 1.08 | 2.28E-05 | Catalyzed the activation, as well as the inactivation of thyroid hormon |
| *Gstm7* | 1.01 | 5.72E-05 | ①Anion binding activity; ②glutathione peroxidase activity; ③glutathione transferase activity |
| *Gstm1* | 1 | 8.40E-05 | Conjugation of reduced glutathione to a wide number of exogenous and endogenous hydrophobic electrophiles |
| Down -regulation | | | |
| *Scd* | -4.51 | 5.20E-06 | Involved in fatty acid biosynthesis and body energy homeostasis |
| *Loc100362027* | -1.72 | 1.95E-04 | Synthesis of proteins in the cell |
| *Rps16* | -1.65 | 1.14E-05 | Synthesis of proteins in the cell |
| *Sds* | -1.63 | 1.74E-04 | ①Involved in metabolizing serine and glycine; ②metabolized threonine to NH4+ and 2-ketobutyrate |
| *Rpl34* | -1.41 | 2.24E-04 | Involved in peptide chain elongation and rRNA processing in the nucleus and cytosol |
| *Sult2a1* | -1.39 | 1.73E-07 | ①Involved in bile acid metabolism; ②steroid and lipid homeostasis |
| *Amdhd1* | -1.37 | 1.32E-04 | Involved in histidine catabolic process |
| *Hal* | -1.24 | 1.71E-04 | Histidine ammonia-lyase activity and ammonia-lyase activity |
| *Eif4a2* | -1.09 | 8.56E-06 | ①Involved in cap recognition; ②negative regulation of RNA-directed 5'-3' RNA polymerase activity |
| *Loc100360449* | -1.72 | 9.43E-05 | / |

## Supplementary Table S14. The top 10 PP and PN peak-genes. (Ranked by Correlation).

| **Gene symbol** | **Position (Chr: Start-End)** | **Correlation** | **Log_2_FoldChange in RNA-Seq** | ***P*-value in RNA-Seq** | **Log_2_FoldChange in ChIP-Seq** | ***P*-value in ChIP-Seq** |
| --- | --- | --- | --- | --- | --- | --- |
| **PP peak-genes** | | | | | | |
| *Rps10l1* | 6:72457379-72467001 | 0.99 | 2.04 | 2.27E-01 | 0.48 | 1.08E-01 |
| *Tmem117* | 7:126685215-126686637 | 0.99 | 1.1 | 8.78E-02 | 0.52 | 1.22E-01 |
| *Rpl39* | 15:19779815-19781023 | 0.99 | 1.18 | 6.45E-02 | -0.03 | 9.33E-01 |
| *B630019k06rik* | X:13761957-13763990 | 0.99 | 1.03 | 2.28E-01 | 0.8 | 4.88E-02 |
| *Sytl1* | 5:144840817-144847246 | 0.99 | 1.15 | 3.83E-01 | 0.07 | 8.42E-01 |
| *Ankrd65* | 5:165824364-165829559 | 0.98 | 1.65 | 9.62E-02 | 0.05 | 8.84E-01 |
| *Klhl31* | 8:78239224-78248969 | 0.98 | 1.49 | 4.66E-01 | 0.03 | 9.19E-01 |
| *Msi1* | 12:40895043-40896566 | 0.98 | 1.41 | 3.74E-01 | 0.84 | 8.60E-03 |
| *Cd79a* | 1:80578670-80585199 | 0.98 | 1.37 | 3.06E-01 | 0.37 | 1.26E-01 |
| *Rnf227* | 10:53579725-53583106 | 0.98 | 2.29 | 3.38E-01 | 0.39 | 1.54E-01 |
| **PN peak-genes** | | | | | | |
| *Dnali1* | 5:136847803-136848061 | 1 | -1.44 | 7.05E-01 | -0.54 | 3.70E-01 |
| *Tmem169* | 9:73029981-73030567 | 1 | -2.43 | 5.16E-01 | 1.34 | 2.49E-01 |
| *Fcrlb* | 13:83457526-83457995 | 1 | -1.33 | 7.27E-01 | 0.49 | 3.53E-01 |
| *Prss54* | 19:9972800-9974910 | 1 | -3.24 | 3.83E-01 | -0.26 | 3.87E-01 |
| *Aabr07043598.1* | 19:26974139-26974708 | 1 | -2.3 | 3.88E-01 | -2.64 | 3.20E-06 |
| *Slc7a15* | 6:31737205-31737433 | 1 | -3.76 | 2.48E-01 | 2.66 | 5.51E-02 |
| *Skor1* | 8:64335689-64336052 | 0.99 | -1.45 | 7.03E-01 | -0.03 | 9.51E-01 |
| *Rnf24* | 3:118222184-118223792 | 0.99 | -1.34 | 6.85E-01 | -0.03 | 9.33E-01 |
| *Fscn2* | 10:104717782-104720394 | 0.99 | -1.83 | 6.28E-01 | -0.04 | 8.77E-01 |
| *Sohlh2* | 2:138825441-138827334 | 0.99 | -2.04 | 5.88E-01 | -0.9 | 2.32E-02 |

**Supplementary Table S15. Nutritional comparison of normal diet and high-fat diet.**

| **Nutritional Index** | **XTCON50H**  **(ND)** | **XTHF45**  **(HFD)** |
| --- | --- | --- |
| Total energy from protein (%) | 20.10 | 20.10 |
| Total energy from fat (%) | 10.00 | 45.10 |
| Total energy from carbohydrate (%) | 69.90 | 34.70 |
| Mass percentage of crude protein (%) | 19.20 | 23.70 |
| Mass percentage of crude fat (%) | 4.30 | 23.60 |
| Mass percentage of carbohydrate (%) | 66.80 | 40.90 |
| Fat source | Soybean oil | Lard (main)+ Soybean oil |
| Sucrose content | Matched to XTHF45 on energy basis | Matched to XTCON50H on energy basis |
| Mineral premix | Standard mineral mix S10026B | Standard mineral mix S10026B |
| Vitamin premix | Standard vitamin mix V10001C | Standard vitamin premix matching Standard vitamin mix V10001C |
| Sterilization method | Gamma irradiation | Gamma irradiation |

## Supplementary Table S16. Primers for RT-qPCR.

| **Primer** | **Sequence5’→3’** |
| --- | --- |
| Human *ACTIN* | F: CATGTACGTTGCTATCCAGGC |
|  | R: CTCCTTAATGTCACGCACGAT |
| Human *CEBPB* | F: CAAGCACAGCGACGAGTACAAGATCC |
|  | R: GCTTGAACAAGTTCCGCAGGGTG |

## Supplementary Table S17. Sequences of siRNAs and primers for cloning, ChIP assays.

| **Gene** | **Application** | **Sequence** | **Species** |
| --- | --- | --- | --- |
| si-*CEBPB*-1 | Cell transfection | CCUCGCAGGUCAAGAGCAA | Human |
| si-*CEBPB*-2 | Cell transfection | CCAAGAAGACCGUGGACAA | Human |
| si-*AHR*-1 | Cell transfection | GGCAUAGAGACCGACUUAATA | Human |
| si-*AHR*-2 | Cell transfection | GUCCCAUAUCCGAAUGAUUAA | Human |
| *AHR*-clone-F | plasmid construction | CTAGCTAGCATGAACAGCAGCAGCGCC | Human |
| *AHR*-clone-R | plasmid construction | CTAGCTAGCATGGCCTCGGGCGACACCC | Human |
| *CEBPB* promoter-clone-F | plasmid construction | CTAGCTAGCTCCCAATCCCGGGGCGGC | Human |
| *CEBPB* promoter-clone-R | plasmid construction | CCGCTCGAGTTACTGCCCCCAAAAGGC | Human |
| *CEBPB* promoter-mut-clone-F | plasmid construction | CAACCGAAGCTTAACTGTCA | Human |
| *CEBPB* promoter-mut-clone-R | plasmid construction | TGACAGTTAAGCTTCGGTTG | Human |
| *CEBPB* promoter-F | ChIP-qPCR | GACTGACGCAACCCACG | Human |
| *CEBPB* promoter-R | ChIP-qPCR | CGGAAAAGATTCCCAAA | Human |
| *CEBPB* enhancer-F | ChIP-qPCR | ACTGCAAGCGGAAGGAGGAGGC | Human |
| *CEBPB* enhancer-R | ChIP-qPCR | GAGGAGGACGTGGAGAGGCTCCCGC | Human |

**Supplementary Table S18. Summary of Cur administration protocols in included clinical trials**

| **Study** | **Formulation** | **Daily dose** | **Dosing frequency** | **Intervention duration** | **Administration route** |
| --- | --- | --- | --- | --- | --- |
| He 2024 | Unspecified | 500 mg | Once daily | 24 weeks | Oral (capsule) |
| Mirhafez 2021 | Phytosomal curcumin | 250 mg | Once daily | 8 weeks | Oral (capsule) |
| Mirhafez 2021 (2) | Curcumin + piperine | 500 mg | Once daily | 8 weeks | Oral (capsule) |
| Hariri 2020 | Phytosomal curcumin | 250 mg | Once daily | 8 weeks | Oral (capsule) |
| Mirhafez 2019 | Phytosomal curcumin | 250 mg | Once daily | 8 weeks | Oral (capsule) |
| Panahi 2017 | Phytosomal curcumin | 1000 mg | Twice daily | 8 weeks | Oral (capsule) |
| Rahmani 2016 | Amorphous curcumin | 500 mg | Once daily | 8 weeks | Oral (capsule) |
